# Knockdown of heat shock protein family D member 1 (HSPD1) promotes proliferation and migration of ovarian cancer cells via disrupting the stability of mitochondrial 3-oxoacyl-ACP synthase (OXSM)

**DOI:** 10.1186/s13048-023-01156-8

**Published:** 2023-04-22

**Authors:** Yaoyun Duan, Juan Yu, Miaojuan Chen, Qinsheng Lu, Fen Ning, Xiaowen Gan, Hanbo Liu, Yixin Ye, Shenjiao Lu, Gendie E. Lash

**Affiliations:** 1 Division of Uterine Vascular Biology, Guangdong Provincial Clinical Research Center for Child Health, Guangzhou Institute of Pediatrics, Guangzhou Women and Children’s Medical Center, Guangzhou Medical University, 510623 Guangzhou, China; 2 School of Medicine, South China University of Technology, Guangzhou, China

**Keywords:** HSP60, OXSM, Lipoic acid, Ovarian cancer

## Abstract

**Background:**

Heat shock protein 60 (HSP60) is essential for the folding and assembly of newly imported proteins to the mitochondria. HSP60 is overexpressed in most types of cancer, but its association with ovarian cancer is still in dispute. SKOV3 and OVCAR3 were used as experimental models after comparing the expression level of mitochondrial HSP60 in a normal human ovarian epithelial cell line and four ovarian cancer cell lines.

**Results:**

Low HSPD1 (Heat Shock Protein Family D (HSP60) Member 1) expression was associated with unfavorable prognosis in ovarian cancer patients. Knockdown of HSPD1 significantly promoted the proliferation and migration of ovarian cancer cells. The differentially expressed proteins after HSPD1 knockdown were enriched in the lipoic acid (LA) biosynthesis and metabolism pathway, in which mitochondrial 3-oxoacyl-ACP synthase (OXSM) was the most downregulated protein and responsible for lipoic acid synthesis. HSP60 interacted with OXSM and overexpression of OXSM or LA treatment could reverse proliferation promotion mediated by HSPD1 knockdown.

**Conclusions:**

HSP60 interacted with OXSM and maintained its stability. Knockdown of HSPD1 could promote the proliferation and migration of SKOV3 and OVCAR3 via lowering the protein level of OXSM and LA synthesis.

**Supplementary Information:**

The online version contains supplementary material available at 10.1186/s13048-023-01156-8.

## Introduction

Ovarian cancer (OC) is known as “the silent killer”. Because of its typically ambiguous symptoms, which make it difficult to treat on a curative basis, it is rarely diagnosed until it is at an advanced stage [1, 2]. Ovarian cancers are divided into epithelial ovarian cancers (EOCs) and non-epithelial cancers. EOCs make up 90% of cases and have serous, mucinous, endometrioid, clear cell (CC), transitional, squamous, mixed, and undifferentiated patterns of histopathology, which make it a complicated process to reveal the mechanism of ovarian cancer pathogenesis and discover effective treatments [3].

Mitochondria play an important role in cancer development through regulating a variety of signal pathways including energy production, oxidative stress, metabolic signaling and apoptotic pathways [4]. Even when oxygen is available, cancer cells have an altered metabolism that result in an increase in lactate production and anaerobic glucose metabolism [5, 6]. As the powerhouse of the cell the major function of mitochondria is ATP production. Beyond energy, tumor cells also need newly synthesized macromolecules to increase their fitness and maintain a redox balance. In addition to glucose-dependent metabolism, tumor cells also utilize alternative oxidizable substrates, such as glutamine, serine, and fatty acids (which serve as anaplerotic sources for tricarboxylic acid (TCA) cycle intermediates) [7].

HSP60, encoded by the nuclear gene HSPD1, belongs to a conserved mitochondrial chaperone family, locating in mitochondria and forming a large multi-subunit complex with its co-chaperonin HSP10 to facilitate proper mitochondrial protein folding [8]. HSP60-dependent mitochondrial proteins exhibit low stability with HSPD1 knockdown [9]. Expression levels of HSP60 in most cancers is higher than corresponding normal tissue and closely related to a poor prognosis [10–15]. On the other hand, the expression of HSP60 is low or unclear in a few cancer types. HSP60 is decreased in clear cell renal cell carcinoma (ccRCC) compared to associated pericarcinous tissues and HSPD1 knockdown promotes ccRCC progression [16, 17]. In the esophageal squamous cell carcinoma (ESCC) [18, 19], hepatocellular carcinoma (HCC) [20–23] and bladder cancer [24, 25], there are contradictory descriptions regarding the role of HSP60 in cancer progression in different studies. As to the association between HSPD1 expression status and the prognosis of patients, there are also conflicting reports in ovarian cancer studies. Studies show that high HSP60 expression is associated with shorter overall survival (OS) and interfering with the expression of HSP60 inhibits the growth of ovarian cancer [26–28]. However, two other studies using immunohistochemistry and analyzing The Cancer Genome Atlas (TCGA) datasets using the Gene Expression Profiling Interactive Analysis 2 (GEPIA2) tool (https://gepia2.cancer-pku.cn/) show that HSP60 expression is associated with a significantly better prognosis [29, 30]. Altogether, these findings suggest that HSP60 could function as bona fide tumor suppressors or oncogenes in a cellular context-dependent manner.

Given the reported conflicting roles of HSP60 in OC, the specific function of HSP60 in the development of ovarian cancer warrants further investigation and it is necessary to reexamine its regulatory role in additional OC cell lines rather than A2780 [27] and TOV-21G [28]. In this study, we investigated the role of HSP60 in the proliferation and migration of SKOV3 and OVCAR3 cell lines. We explored differentially expressed proteins via proteomics and validated the interaction between HSP60 and OXSM. Therefore, we speculated that knockdown of HSP60 promoted the proliferation and migration of SKOV3 and OVCAR3 cells through disrupting the stability of OXSM and lipoic acid synthesis. This finding will help us to deepen our understanding of HSP60 and provide more accurate scientific basis for the anticancer strategy.

## Materials and methods

### Cell cultures

The normal human ovarian epithelial cell line (IOSE80) was purchased from Shanghai Fuheng Biotechnology (Shanghai, China). The human ovarian cancer cell lines (SKOV3, OVCAR3, ES2, A2780) were kindly provided by Procell Life Science & Technology Co., Ltd (Wuhan, China). Cells were cultured in the appropriate culture medium according to the instructions and were cultured at 37℃ in a humidified atmosphere with 5% CO_2_. Human cells were short tandem repeat (STR)-profiled, used between passages 3 and 20, and regularly examined for mycoplasma.

### Databases of Kaplan–Meier plotter

Kaplan–Meier plots (https://kmplot.com/analysis/) combine the bigger amount of genes’ impact on the visualization of patients with cancer, through TCGA, European Genome-phenome Archive (EGA) and Gene Expression Omnibus (GEO) databases. In this study, the prognostic value of the mRNA expression of HSPD1 and OXSM were evaluated using the Kaplan–Meier plotter. The progression-free survival (PFS) of patients with OC were determined by dividing the patient samples into two groups based on median expression (high vs. low expression) and assessing using a Kaplan–Meier survival plot, with a hazard ratio with 95% confidence intervals and log rank *P*-value.

### Ovarian cancer tissue array analysis of HSP60 protein levels

The ovarian cancer tissue microarray was purchased from the Shanghai Outdo Biotech Company (Shanghai, China). The serial number was HOvaC070PT01. This microarray included 65 ovarian cancer tissue samples and 5 adjacent normal epithelial tissues samples. Immunohistochemistry to detect HSP60 was performed using anti-HSP60 antibody (Proteintech, Wuhan, China; dilution 1:200). The immunohistochemistry (IHC) staining intensity was graded from 0 to 3 (0, negative; 1, weak; 2, moderate; and 3, strong). The staining quantity was graded from 1 to 4 (1, < 25%; 2, 25–50%; 3, 50–75%; and 4, > 75%) in the tissue array. The staining scores were calculated by multiplying the staining intensity with the staining quantity.

### Plasmid DNA and siRNA transfection

The cells (SKOV3 1 × 10^5^, OVCAR3 1.2 × 10^5^) were seeded into 6-well plates. siRNAs were purchased from Shanghai GenePharma Co., Ltd (Shanghai, China). After 24 hours, cells were transfected with HSPD1 siRNA (si-HSPD1), OXSM siRNA (si-OXSM) or negative siRNA (si-NC) using Lipofectamine® 3000 (ThermoFisher Scientific, MA, USA) following the manufacturer’s protocol. OXSM plasmid (pRP[Exp]-EGFP/Puro-CAG > hOXSM ) and corresponding empty vector were purchased from VectorBuilder (Guangzhou, China). The siRNA sequences targeting the human HSPD1 gene, the human OXSM gene, and the negative control (NC) sequence were as follows: 5’-GTTGCAAAGTCAATTGACT-3’ (si-HSPD1-1), 5’-GTTGCTACGATTTCTGCAA-3’ (si-HSPD1-2), 5’-GGAATCATTGACCCAAC, AA-3’ (si-HSPD1-3), 5’-GGTCAGCATTCGATATAAA-3’ (si-OXSM-1), 5’-GGA, GACTCATTTAGATTTA-3’ (si-OXSM-2), 5’-TTCTCCGAACGTGTCACGT-3’ (si-NC).

### Lentiviral packaging and stable cell lines construction

A lentiviral plasmid (pLKD-CMV-Puro-U6-shRNA) was purchased from Obio Technology (Shanghai, China). The shRNA sequences targeting the human HSPD1 gene (Gene ID: 3329) and the negative control (NC) sequence were as follows: 5’-GGAATCATTGACCCAACAA-3’ (sh-HSPD1) and 5’-TTCTCCGAACGTGTCACGT-3’ (sh-NC). When the cells reached 30% confluence in six-well plates, lentiviral solution was added with a final concentration of 5 µg/ml polybrene. The medium was replaced with fresh culture medium after 48 h transfection. A stable transfected cell line was established by puromycin selection (5 µg/ml for SKOV3 and 2 µg/ml for OVCAR3). Transfection efficiency was evaluated using qRT-PCR and western blotting to validate HSPD1 knockdown.

### Proliferation using the real-time cell analysis (RTCA) xCELLigence system

The RTCA utilizes specialized plates that contain interdigitated electrodes, which can be used to determine cell proliferation in real time. A total of 2000 SKOV3 cells or 3000 OVCAR3 cells were added to duplicate wells of a CIM-E-Plate, and each experiment repeated on three separate occasions. The RTCA chamber was housed within a cell culture incubator at 37˚C and 5% CO_2_ in air. Recordings were taken every 15 min.

### 5-ethynyl-2’-deoxyuridine (EdU) incorporation assay

Cell proliferation rates were analyzed using the EdU incorporation assay. The analysis was performed using the Cell-Light EdU Apollo 488 In Vitro kit (RiboBio Co., Ltd., Guangzhou, China) following the manufacturer’s protocol.

### Soft agar colony assay

8000 cells were seeded in 0.3% top agar in growth medium over a layer of 0.5% agar in a 6-well plate. After 21 days of incubation, the plates were stained with 0.1% Crystal Violet for 2 h. Colonies whose diameter was larger than 0.2 mm were counted. All assays were performed in triplicate.

### Glucose uptake and lactate production

Cell culture medium was collected after cell culture for 3d. Glucose and lactate concentrations in the medium were determined by Glucose Assay Kit-WST (Dojindo, Shanghai, China) and Lactate Assay Kit-WST (Dojindo, Shanghai, China), respectively, in accordance with the manufacturer’s instructions.

### Wound healing assay

Cells were seeded into 24-well dishes. After 70% cell confluence was reached, a pipette tip (200 µL) was used to make a straight scratch with the same width in the middle of the well, followed by washing with phosphate buffer solution (PBS) and replacing with new medium without FBS. Images were taken at t = 0 h and after incubation in 5% CO_2_ at 37℃ for 12 and 24 h, respectively. The area covered by cells was measured and compared with the area at 0 h to quantify the cell migration.

### Transwell assay

Cell migration was measured using polycarbonate membrane boyden chambers. Cells (SKOV3 2 × 10^4^, OVCAR3 5 × 10^4^) were seeded onto the top chamber, and the lower chamber was added with 600 µL medium containing 10% FBS. After incubation for 24 h, a cotton swab was used to remove cells on the top surface of membrane. The migrated cells were fixed with 4% paraformaldehyde and stained with 0.1% crystal violet for 20 min. Finally, the cells were captured from at least 3 random fields.

### Real-time PCR

Briefly, total RNA was extracted with TRIzol reagent (Invitrogen, CA, USA) and cDNA was synthesized using PrimeScript™ RT Master Mix (Takara, Dalian, China). qRT-PCR was performed using TB Green® Premix Ex Taq™ II kit (Takara, Dalian, China) with each sample run in triplicate, and the reaction solution was prepared using the reverse transcription DNA as template according to the following system: TB Green Mix 10µL, forward primer (10 µM) 0.8 µL, reverse primer (10 µM) 0.8 µL, ROX 0.4 µL, DNA template 2.0 µL, ddH2O 6.0 µL. Relative transcript abundance was normalized to 18s. The following primer sequences were used. 18s forward: 5’-CAGCCACCCGAGATTGAGCA-3’, 18s reverse: 5’-TAGTAGCGACGGGCGGTGTG-3’, SUCLG2 forward: 5’-CAAAAGACCCTAATGTTGTGGGA-3’, SUCLG2 reverse: 5’-TTCAGCAACCATCACCTTGTT-3’, OXSM forward: 5’-TGATGCTGGTCACATAACTGC-3’, OXSM reverse: 5’-TCCCAATGGTGTGGAAGTAGC-3’.

### Western blotting

Protein was extracted with RIPA lysis buffer (Beyotime, Shanghai, China). The concentration of total protein in cells was determined by the BCA Protein Assay kit (Beyotime, Shanghai, China) and adjusted for equal loading. Standard procedures were used to run protein samples on SDS-PAGE gels and subsequently transferred to PVDF membranes (Millipore, USA). The following primary antibodies utilized in this study: anti-SDHA antibody (1:1000, Bioworld, Nanjing, China), anti-HSPD1 (1:300, Santa Cruz, Texas, USA), anti-GAPDH (1:5000, Proteintech, Wuhan, China), anti-VDAC1, anti-SUCLG2, anti-ATP5A, anti-MT-CO1 (1:1000, SAB, Nanjing, China), anti-OXSM (1:2000, Genetex, CA, USA). Blots were visualized using ECL reagents (Pierce, IL, USA) by a chemiluminescence imaging system (Bio-Rad, CA, USA).

### Quantitative proteomics by the Liquid Chromatograph Triple Quadrupole Mass Spectrometer (LC-MS/MS)

SKOV3 cells were transfected with sh-NC or sh-HSPD1 lentiviruses. Stable cell lines were harvested and submitted for proteomic analysis (Shanghai Genechem, Shanghai, China). In brief, cells were lysed by SDT buffer (4% SDS, 100 mM DTT, 150 mM Tris-HCl pH 8.0) and digested via filter-aided sample preparation (FASP Digestion). 100 µg peptide mixture of each sample was labeled using tandem mass tagging (TMT) reagent according to the manufacturer’s instructions (Thermo Fisher Scientific). After TMT labeling, the sample was fractionated with reversed phase (RP) chromatography. Each fraction was injected for LC-MS/MS analysis. A peptide and protein false discovery rate of 1% was enforced using a reverse database search strategy. Proteins with Fold change > 1.2 and *P* value (Student’s t test) < 0.05 were considered to be differentially expressed proteins.

### Kyoto Encyclopedia of genes and genomes (KEGG) enrichment

KEGG Orthology-Based Annotation System (KOBAS) 3.0 [31], a web-based tool for gene and protein functional annotation and pathway enrichment analysis, was used to predict the enriched pathways of these changed genes.

### Co-immunoprecipitation (Co-IP)

Briefly, 1 mg of protein sample was incubated with HSPD1 antibody (1 µg, Proteintech, Wuhan, China) at 4℃ overnight. The next day, 40 µL Protein A + G Agarose beads (Beyotime, Shanghai, China) were added to the protein sample and incubated at 4℃ for 3 h. The beads were pelleted and washed with protein lysis buffer 5 times. Finally, the bead pellet was re-suspended in 1×loading buffer, boiled, and loaded onto SDS-PAGE.

### Proximity ligation assay (PLA)

PLA was conducted using the Duolink Detection kit (Sigma, MO, USA), as per the manufacturer’s instructions. Cells (20,000–30,000 cells) were cultured in an 8-well chamber slide. Cells were fixed and permeabilized. Cells were incubated in the blocking buffer for 1 h and incubated at 4℃ overnight with a pair of anti-HSPD1 (Santa Cruz, Texas, USA) and anti-OXSM (Genetex, CA, USA) antibodies. The next day, cells were incubated with secondary proximity probes (Duolink In Situ PLA probe anti-rabbit plus, DUO92002 and anti-mouse minus, DUO92004). Then, samples were incubated with the Detection Reagents Red (DUO92008) containing T4 DNA ligase (1 unit/µL) and amplification solution (DNA polymerase, 10 units/µL) followed by counterstaining with DAPI. Fluorescence signals were detected (63× objective) by confocal microscopy (SP8, Leica).

### Mitochondria preparation

Intact mitochondria were isolated from cultured cells with a mitochondria isolation kit (ThermoFisher Scientific, MA, USA) according to the manufacturer’s instructions. The resultant mitochondria pellet was lysed for western blotting.

### Statistical analysis

All experiments were conducted at least for three times and data shown as mean ± standard deviation (SD). GraphPad Prism 8.0 (GraphPad Software, San Diego, CA, USA) was used for statistical analyses and drawing figures. Student’s t test was applied between two groups. Multiple comparisons were conducted using one-way analysis of variance (ANOVA) followed by Dunnett’s test. Differences were considered to be significant at *P* < 0.05.

## Results

### Prognostic significance of HSPD1 expression in ovarian cancer

**Fig. 1:**
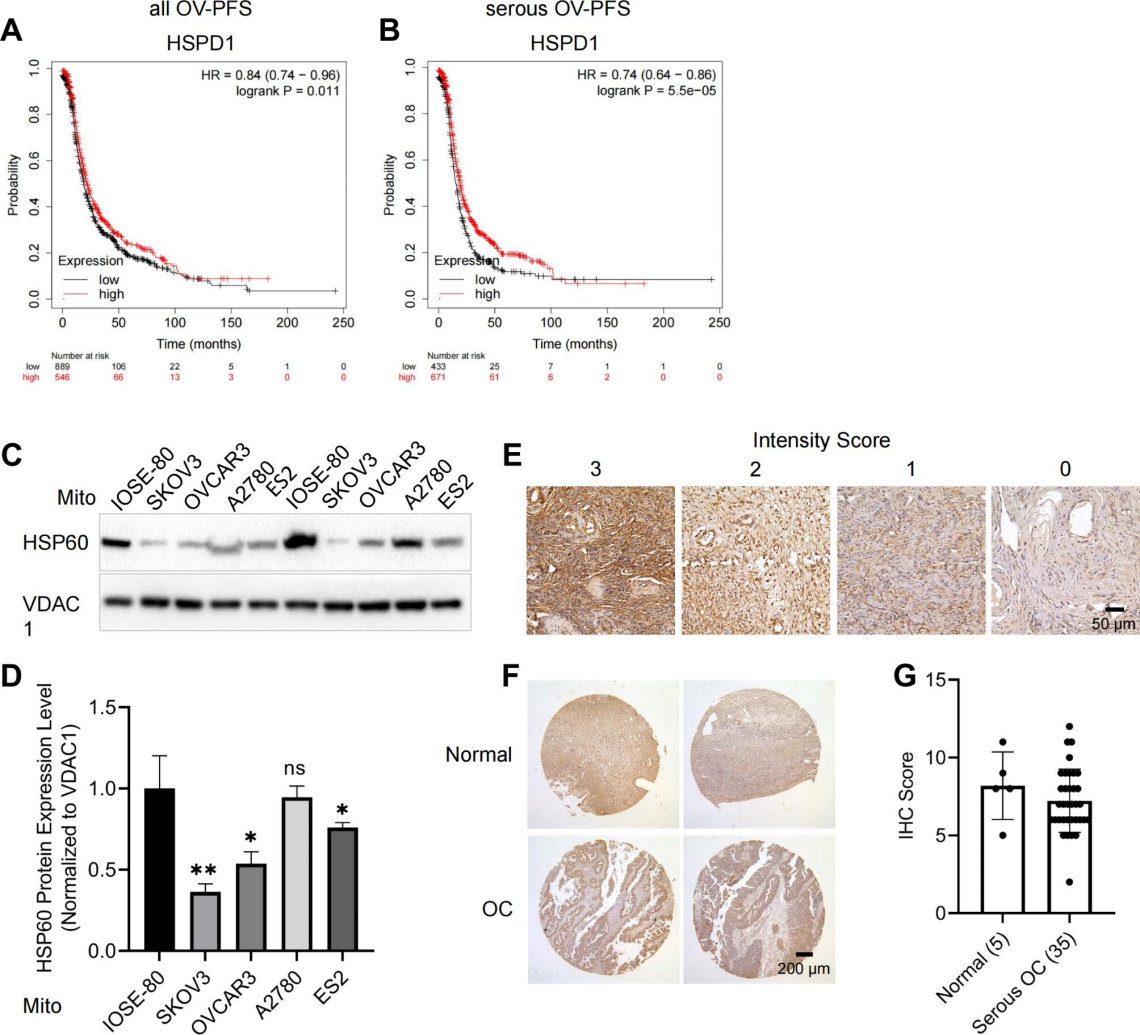
The association of HSP60 with the prognosis of ovarian cancer patients and its expression in human ovarian cancer cell lines and tissues. **A, B** The curve plot for progression-free survival between HSPD1 high and low transcript groups in all OC and serous OC, respectively. The red line indicates patients with high HSPD1 levels and the black line indicates patients with low HSPD1 levels, log rank *P* = 0.01 and 0.00006, respectively. **C** Differential expression of HSP60 in the isolated mitochondria of OC cell lines. **D** Quantification analysis of the protein expression level in the **C**. **E** HSP60 expression was measured by IHC in tissue microarray. Representative images of strong (score 3), medium (score 2), weak (score 1), and negative (score 0) staining are shown. **F** IHC images of HSP60 in normal (upper) and OC (lower) tissues according to tissue microarray. **G** Statistical analysis of the protein expression of HSP60 according to the staining scores of OC and normal tissues. *P < 0.05, **P < 0.01

Earlier studies evaluated the association between HSP60 expression levels and ovarian cancer with inconsistent findings. In the current study, firstly, the databases in Kaplan–Meier plotter were used to demonstrate the relationship between the expression of HSPD1 and prognosis of OC. Low HSPD1 expression was associated with unfavorable prognosis in all OC (PFS: HR = 0.84, *P* = 0.01; Fig. 1A) and serous OC (PFS: HR = 0.74, *P* = 0.00006; Fig. 1B). HSPD1 was mainly located in the mitochondria [32, 33] and this was validated in the OC cell lines (Fig. S1). Furthermore, to explore the role of HSPD1 in ovarian cancer development, we isolated the mitochondria and evaluated the differential expression of HSPD1 by western blot analysis in one normal ovarian epithelial cell line and four ovarian cancer cell lines. The normal human ovarian epithelial cell line (IOSE-80) had significant higher expression of HSPD1 as compared to OC cell lines (SKOV3 *P* = 0.007, OVCAR3 *P* = 0.02, ES2 *P* = 0.04), whereas A2780 showed no significant difference (Fig. 1C, D). We also explored the expression of HSPD1 at the protein level in OC samples using a tissue microarray. Typical staining images with different intensity scores in the tissue array are shown in Fig. 1E. There was no difference in the HSP60 immunoreactivity scores between the normal and OC tissues (*P* = 0.3) (Fig. 1F, G). Opposing roles as a tumor suppressor and promoter may exist in different cell lines with different sources and genomic background. Further experiments carefully designed to decipher the opposing roles will lead to better understanding of OC development.

### Downregulation of HSPD1 promoted the proliferation ability of ovarian cancer cells

**Fig. 2:**
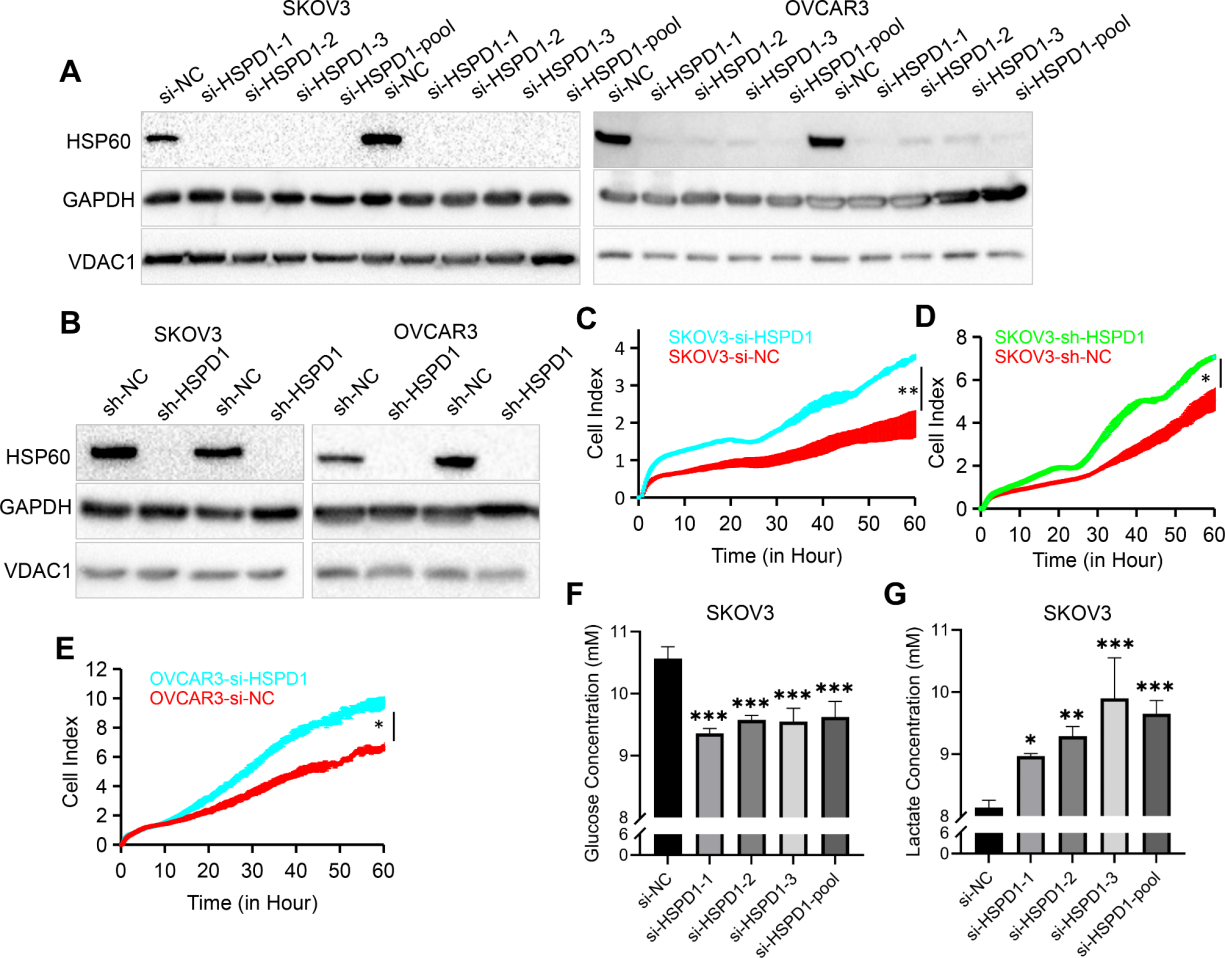
Downregulation of HSPD1 promotes the proliferation ability of ovarian cancer cells. **A** Western blotting analysis of HSPD1 expression in SKOV3 and OVCAR3 cells transfected with NC siRNA and HSPD1 siRNA, respectively. **B** SKOV3 and OVCAR3 cells were transduced with lentiviruses delivering negative control shRNA (sh-NC) or HSPD1 shRNA (sh-HSPD1). Changes in HSPD1 protein expression was measured by western blotting. **C** Effects of HSPD1 knockdown with siRNA on the proliferation of SKOV3 analyzed by the RTCA assay. **D** Effects of HSPD1 knockdown with lentivirus shRNA on the proliferation of SKOV3 analyzed by the RTCA assay. **E** Effects of HSPD1 knockdown with siRNA on the proliferation of OVCAR3 analyzed by the RTCA assay. **F** Graphical representation of the concentration of glucose in cell culture medium after 72 h cell culture. **G** Graphical representation of the concentration of lactate in cell culture medium after 72 h cell culture. *P < 0.05, ** *P* < 0.01 and ***P < 0.001

siRNA-mediated and lentiviral vector-mediated transfection were used to determine the effect of HSPD1 knockdown in ovarian cancer cells. Western blotting analysis was performed to confirm the knockdown efficiency. GAPDH and mitochondrial protein -VDAC1 were used as the protein loading control at the same time. They showed no difference between the HSPD1 knockdown and control group (Fig. 2A, B), which indicated both GAPDH and VDAC1 could be chosen as loading control proteins. We used 3 siRNAs targeting different domains of the HSPD1 mRNA sequence to transfect SKOV3 and OVCAR3 cells to avoid off-target effects. HSP60 protein levels were all drastically reduced (Fig. 2A), so the siRNA pool (si-HSPD1) was used for subsequent experiments. To sustain long-term repression of HSPD1 gene, we also constructed the knockdown stable cell lines. HSP60 protein expression was evaluated by western blotting, and the results showed that HSP60 levels were markedly reduced by LV-shRNAs in both SKOV3 and OVCAR3 cells (Fig. 2B). The functional effect of HSPD1 knockdown on SKOV3 and OVCAR3 cell proliferation was evaluated by RTCA assays. The results showed that downregulation of HSPD1 via siRNA or LV-shRNA significantly promoted the proliferation ability of ovarian cancer cells (*P* = 0.003, 0.03, 0.02, respectively) (Fig. 2C-E). Cell proliferation was also assessed immunocytochemically using EdU incorporation. Compared with the control group, HSPD1 knockdown group showed increased cell proliferation indicated by higher EdU positive ratio (*P* = 0.00008) (Fig. S2A, C). To further explore the role of HSPD1 in tumorigenesis, soft agar colony formation assay was performed. HSPD1 knockdown cells formed more colonies than control cells (*P* = 0.02) (Fig. S2B, D). It was observed that glucose levels in the culture medium of the HSPD1 knockdown cells were lower than those of the control cells (si-HSPD1-1 *P* = 0.0006, si-HSPD1-2 *P* = 0.0007, si-HSPD1-3 *P* = 0.0008, si-HSPD1-pool *P* = 0.0008 compared with si-NC) (Fig. 2F). Indeed, lactate concentrations were elevated in HSPD1 knockdown medium compared to controls (si-HSPD1-1 *P* = 0.03, si-HSPD1-2 *P* = 0.002, si-HSPD1-3 *P* = 0.0008, si-HSPD1-pool *P* = 0.0007 compared with si-NC) (Fig. 2G). The results indicated that downregulation of HSP60 enhanced glycolysis leading to Warburg metabolic phenotype, which was consistent with the increased proliferation.

### Repression of HSPD1 promoted ovarian cancer cell migration in vitro

**Fig. 3:**
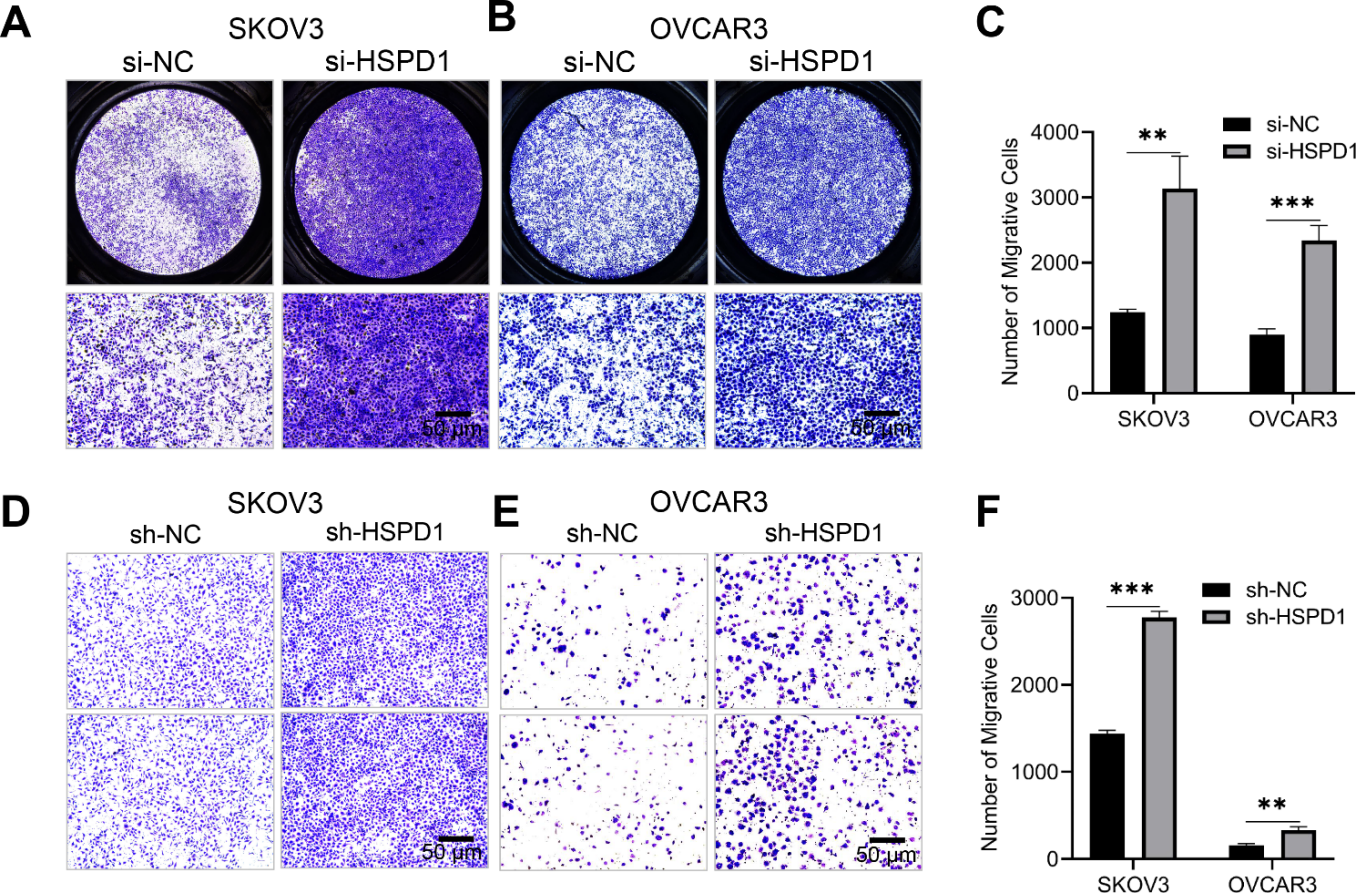
Knockdown of HSPD1 promoted ovarian cancer cell migration in vitro. **A, B** Effects of HSPD1 knockdown with siRNA on the migration of SKOV3 and OVCAR3 analyzed by transwell assay. The upper row are merged pictures. Scale bar = 50 μm. **C** Quantification of migrative cells in the si-NC and si-HSPD1 group. **D, E** Effects of HSPD1 knockdown with lentivirus shRNA on the migration of SKOV3 and OVCAR3 analyzed by transwell assay. Scale bar = 50 μm. **F** Quantification of migrative cells in the sh-NC and sh-HSPD1 group. *P < 0.05, **P < 0.01 and ***P < 0.001

Transwell assays and wound healing tests were conducted to find the role of HSP60 in OC cell migration. Following HSPD1 knockdown with siRNA transient transfection, the number of cells in the si-HSPD1 group migrated from the upper chamber to the lower membrane was significantly increased compared with si-NC group for both SKOV3 (*P* = 0.002) and OVCAR3 (*P* = 0.0007) cell lines (Fig. 3A-C). Similarly, in wound healing tests, the results showed that knockdown of HSPD1 increased migration of SKOV3 at 12 h (*P* = 0.0008) and 24 h (*P* = 0.006) (Fig. S3A, B). Furthermore, HSPD1 knockdown stable cell lines (sh-HSPD1) also showed increased migration capacities compared with cells transfected with empty vector lentivirus (sh-NC) (Fig. 3D-F) The results suggested that HSP60 may inhibit the migration of specific ovarian cancer cell.

### Proteomic analysis of differentially expressed proteins between HSPD1 knockdown and control cells

**Fig. 4:**
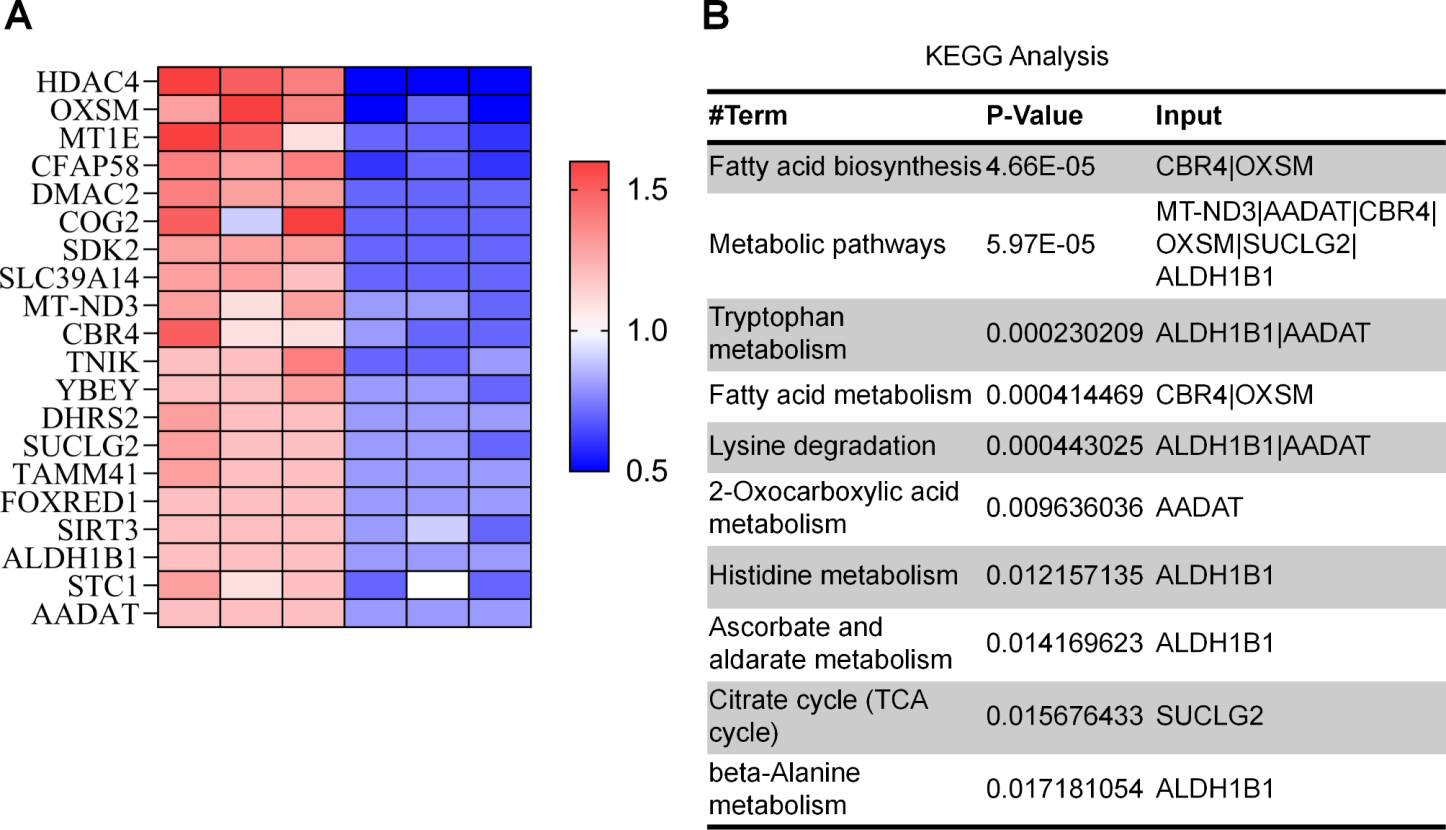
Identification of differentially expressed proteins in HSP60 knockdown SKOV3 cells via proteomics. **A** Heat map of Top 20 downregulated differentially expressed proteins after HSP60 knockdown. **B** KEGG enrichment pathways of the Top 20 downregulated proteins

To further understand the effects of HSPD1 deletion on ovarian cancer cells, we performed quantitative proteomic analysis. Among 591 differentially expressed proteins, we selected the top 20 downregulated proteins according to the log ratio expression values (Fig. 4A). To explore the functions of HSP60 and the related pathways, KEGG analysis was performed to determine possible enrichment functions and pathways. The result showed that the top 5 pathways were fatty acid biosynthesis, metabolism pathways, tryptophan metabolism, fatty acid metabolism and lysine degradation (Fig. 4B). OXSM and CBR4 were responsible for two important steps in the fatty acid synthesis pathway, and OXSM was the second most downregulated protein and involved in 3 of the 5 enriched KEGG pathways. The results revealed that OXSM might alter the biological behaviors of OC cells by modulating fatty acid biosynthesis and metabolism pathways.

### HSP60 knockdown disrupted the stability of mitochondrial protein-OXSM and lipoic acid synthesis

**Fig. 5:**
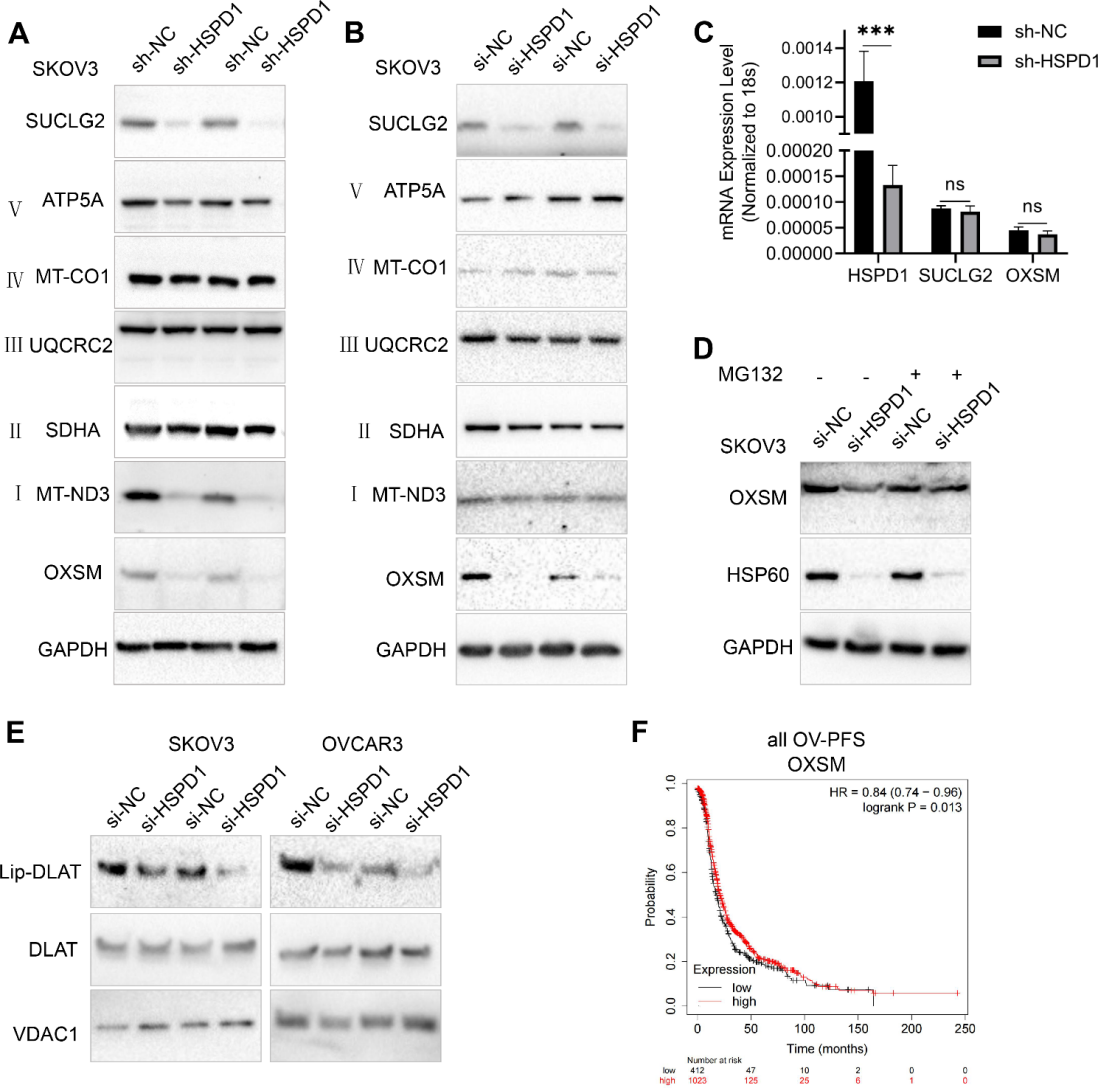
HSP60 knockdown disrupted mitochondrial protein-OXSM stability and lipoic acid synthesis. **A, B** Western blotting analysis was used to validate expression changes of individual mitochondrial proteins in HSP60 knockdown SKOV3 cells with siRNA and lentivirus shRNA, respectively. **C** Quantitative RT-PCR analysis was used to analyze the mRNA levels of HSPD1, SUCLG2 and OXSM. **D** SKOV3 cells were transfected with si-NC and si-HSPD1, 48 h later, cells were treated with or without 0.2 µM MG132 for 24 h prior to cell lysis. The level of OXSM protein was examined by western blotting. **E** Western blotting of lipoylated proteins (lip-DLAT) and total DLAT from extracts of OC cells with HSPD1 knockdown. **F** Kaplan-Meier survival analysis of progression-free survival between OXSM high and low transcript groups in all OC. The red line indicates patients with high OXSM levels and the black line indicates patients with low OXSM levels, log rank *P* = 0.01. ***P < 0.001

Western blotting analysis was further performed to validate the reliability of the proteomic data. Protein levels of OXSM were significantly decreased with HSPD1 knockdown in OC cells (Fig. 5A, B). Oxidative phosphorylation provides most of the ATP to support life and is responsible for setting and maintaining metabolic homeostasis [34]. Oxidative phosphorylation relies on the electron transport chain (ETC) complex. Among those components of the mitochondrial electron transport chain identified by proteomics, the most differentially expressed proteins belonged to ETC complex I (Table S1). Western blotting analysis showed that MT-ND3 was downregulated after knockdown of HSPD1 with lentivirus but not with siRNA transfection (Fig. 5A, B). Meanwhile, SDHA (ETC complex II), UQCRC2 (ETC complex III), MT-CO1 (ETC complex IV) and ATP5A (ETC complex V) showed no significant difference between HSPD1 knockdown and control groups (Fig. 5A, B). The TCA cycle needs to generate substrates that enter the ETC for oxidative phosphorylation. SUCLG2 is one important enzyme in the TCA cycle, coupling the hydrolysis of succinyl-CoA to the synthesis of GTP. The protein level of SUCLG2 was significantly decreased in the HSPD1 knockdown group, which was also consistent with the proteomic analysis (Fig. 5A, B). We further investigated the mechanisms underlying the downregulation of mitochondrial proteins by HSP60 deletion. mRNA levels of SUCLG2, OXSM genes were not significantly altered after knockdown of HSPD1 (HSPD1 *P* = 0.00008, SUCLG2 *P* = 0.2, OXSM *P* = 0.09) (Fig. 5C), suggesting that HSP60 suppression in OC cells did not change the transcription profile of these genes. HSP60 is essential for the folding and assembly of newly imported proteins in the mitochondria [33]. Therefore, we hypothesized that HSP60 knockdown could induce increases in unfolded or misfolded proteins, protein stability loss and subsequent protein degradation. MG132 (carbobenzoxy-Leu-Leu-leucinal) is a peptide aldehyde, which effectively blocks the proteolytic activity of the 26 S proteasome complex [35]. As a proteasome inhibitor, it is usually utilized to rescue proteins from degradation [36]. It was found that MG132 restored OXSM expression in HSP60-deficient cells (Fig. 5D), indicating that HSP60 depletion induced degradation of OXSM via the proteasome. OXSM is the second enzyme in LA synthesis and the LA synthetic pathway is required to maintain mitochondrial function [37]. LA is required for the catalytic activity of a number of mitochondrial enzymes such as pyruvate dehydrogenase and a-ketoglutarate dehydrogenase [37]. DLAT is a subunit of pyruvate dehydrogenase. Next, it was determined whether HSPD1 knockdown impacted protein lipoylation using a lipoic acid-specific antibody as a measure of DLAT lipoylation. HSPD1 knockdown resulted in loss of protein lipoylation (Fig. 5E), indicating that HSPD1 played an important role in LA synthesis mediated by OXSM. In addition, Kaplan-Meier survival analysis showed that low OXSM expression was associated with unfavorable prognosis in all OC (PFS: HR = 0.84, *P* = 0.01; Fig. 5F).

### HSP60 inhibited the proliferation of OC cells via LA synthesis pathway mediated by OXSM

**Fig. 6:**
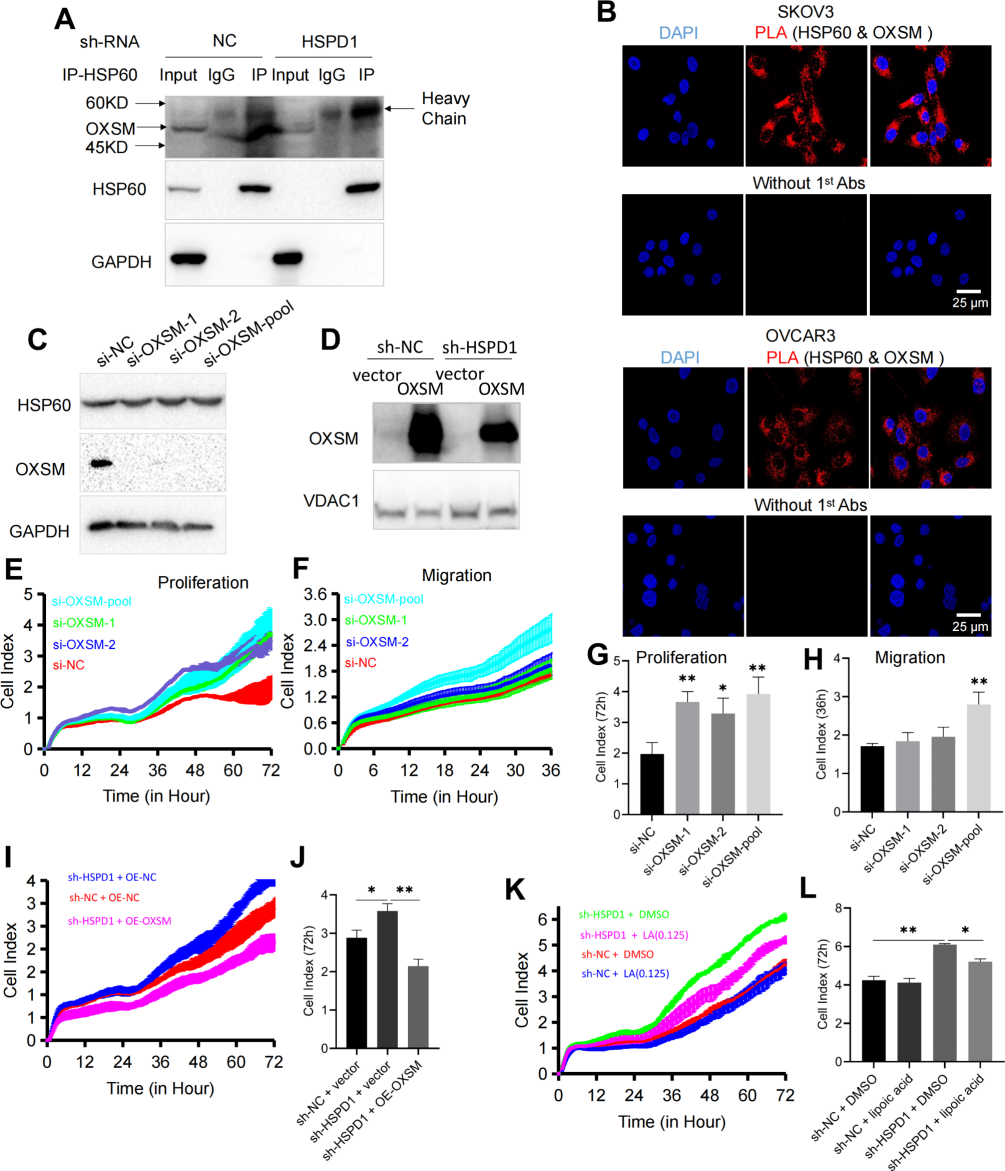
HSPD1 promotes ovarian cancer progression by maintaining function of OXSM. **A** Co-immunoprecipitation of OXSM with HSP60 in SKOV3 cells. **B** Examination of interaction of OXSM-HSP60 PLA signal in SKOV3 and OVCAR3 cells lines. Scale bar = 25 μm. **C** Western blotting analysis of OXSM expression in SKOV3 cells transfected with NC siRNA and OXSM siRNA. **D** Validation of the overexpression of OXSM in OC cells transfected with OXSM plasmid via western blotting. **E, G** Effects of OXSM knockdown with siRNA on the proliferation of SKOV3 analyzed by the RTCA assay. **F, H** Effects of OXSM knockdown on the migration of SKOV3 analyzed by the RTCA assay. **I, J** Cell proliferation was detected by RTCA assay after transfection with sh-HSPD1 and OE-OXSM. Quantification of the cell index after 72 h culture in the RTCA assay. **K, L** SKOV3 cells transfected with lentivirus sh-NC or sh-HSPD1 treated with lipoic acid (0 or 0.125 µM). Quantification of the cell index after 72 h culture in the RTCA assay. *P < 0.05, **P < 0.01

To explore the mechanism underlying the regulatory role of HSP60 in OC we investigated whether OXSM was the HSP60-interacting mitochondrial protein. We found that HSPD1 interacted with OXSM in SKOV3 cell using Co-IP (Fig. 6A). In this study we also validated the interaction using HSPD1 and OXSM antibodies for PLA. The PLA signals arose in SKOV3 and OVCAR3 cells and there was no signal without primary antibodies (Fig. 6B). It suggested interaction between HSPD1 and OXSM in cells. To investigate the effect of OXSM on ovarian cancer cell proliferation and migration, we performed knockdown on the OXSM gene and overexpressed OXSM using an OXSM overexpression plasmid, respectively. The expression of OXSM was significantly downregulated or enhanced (Fig. 6C, D). To identify changes in SKOV3 cell progression associated with OXSM downregulation, cell proliferation and migration were investigated using RTCA assays. OXSM downregulation markedly increased cell proliferation (si-OXSM-1 *P* = 0.004, si-OXSM-2 *P* = 0.02, si-OXSM-pool *P* = 0.007 at 72 h) (Fig. 6E, G) and migration (si-OXSM-pool *P* = 0.005 at 36 h) compared with controls (Fig. 6F, H). However, OXSM downregulation with si-OXSM-1 and si-OXSM-2 didn’t cause a significantly different effect on migration compared with si-NC cell (si-OXSM-1 *P* = 0.06, si-OXSM-2 *P* = 0.5 at 36 h) (Fig. 6F, H). There were many factors contributing to the inconsistency in resutls. One reason might be the weak inhibitory effects of OXSM on migration. Other possible reasons for the inconsistent effect were that siRNAs against different regions of gene could have different silencing effects or siRNAs targeting alternative splicing generated multiple transcript and protein isoforms from the same gene. We also explored the role of OXSM from another perspective. Functional experiments showed that OXSM overexpression could reverse sh-HSPD1-mediated proliferation promotion in SKOV3 cells (Fig. 6I). HSPD1 knockdown markedly increased cell proliferation (*P* = 0.03 at 72 h) and OXSM overexpression caused decreased proliferation in stable HSPD1 knockdown cells compared with empty vector transfected cells (*P* = 0.006 at 72 h) (Fig. 6J). These data suggested that knockdown of HSPD1 restrained the proliferation of OC cells through targeting OXSM. OXSM is involved in LA synthesis. For the elongation of fatty acid chains to produce LA, OXSM activity is necessary [37]. To further validate the role of OXSM, HSPD1 knockdown stable cell lines were treated with lipoic acid or DMSO. The result showed that lipoic acid could reverse sh-HSPD1-mediated proliferation promotion (Fig. 6K). HSPD1 knockdown markedly increased cell proliferation (*P* = 0.007 at 72 h) and addition of lipoic acid (0.125 µM) resulted in decreased proliferation in stable HSPD1 knockdown cells compared with DMSO treated cells (*P* = 0.02 at 72 h) (Fig. 6L). Collectively, these findings showed that HSPD1 knockdown could inhibit the LA synthesis mediated by OXSM, which led to promotion of ovarian cancer cell proliferation.

## Discussion

Mitochondria can not only function as an “energy factory”, but also signaling hubs with biosynthetic, bioenergetics and signaling functions. Firstly, tumor metabolism not only relies on glucose but also alternative oxidizable substrates, such as glutamine, serine and fatty acids. Functional mitochondria are essential for tumor growth depending on their biosynthetic role [38]. In ovarian cancer, glutamine dependence is correlated with cancer invasiveness and patient survival [39]. The glutaminase inhibitors compound can inhibit cancer progression via reducing glutamine catabolism [40]. Secondly, many studies have shown that abnormalities in mitochondrial enzymes involved in bioenergetics are closely related to tumorigenesis. Enhanced citrate synthase activity in pancreatic cancer can promote the conversion of glucose to lipids, thereby maintaining pancreatic cancer’s metabolism [41]. Isocitrate dehydrogenase mutations in cancer promote the development of a neomorphic enzyme that converts isocitrate into D-2-hydroxyglutarate, a metabolite with oncogenic effects and epigenetic regulation [42]. Thirdly, mitochondria are the major source of intracellular ROS. In most cases, mitochondrial ROS (mtROS) is considered to activate different potentially oncogenic signaling pathways [43, 44]. However, Vitamin K3 has the potential to be developed as an antitumor agent since it induces apoptosis by increasing the generation of ROS in ovarian cancer cells [45]. Previous studies provide an insight into how mitochondria-related molecules function in cancer. Numerous drugs have been proposed to date for the treatment of cancer that target various aspects of mitochondrial metabolism (bioenergetics, signaling, and biosynthesis) [7]. In conclusion, targeting mitochondrial function could be another promising anticancer strategy.

Mitochondrial protein HSP60 is the key chaperonin that controls mitochondrial proteostasis. The roles of HSP60 in tumorigenesis and progression have been context dependent [30]. HSP60 was overexpressed in a large number of tumors and promoted the progression of cancer. HSP60 can inhibit apoptosis of cancer cells via stabilizing survivin [21, 46, 47], curbing p53 function [46], and antagonizing the pro-apoptotic function of Cyclophilin D [48]. HSP60 promotes the proliferation of cancer cells via supporting Erk1/2 activation [49]. On the other hand, HSP60 can also function as tumor suppressor. HSP60 can induce apoptosis through promoting the maturation of caspase 3 [50], interacting with a tumor suppressor - fragile histidine triad protein (FHIT) [51]. In addition, HSP60 plays opposing roles as a promoting and suppressing molecule within the same cancer type. In hepatocellular carcinoma, HSP60 sustains cell survival via stabilizing survivin [21]. However, it induced cell differentiation and inhibited cell invasion and migration [20]. Oxidation of HSP60 mediates PKCδ-mtROS-triggered mitogen-activated protein kinase (MAPK) activation to cause suppressed proliferation [52]. Further carefully designed experiments are needed to unravel these contradictory findings and obtain a better understanding of cancer development.

As to HSP60 in ovarian cancer, one study reported that higher levels of HSP60 expression in ovarian tumors correlated with shorter overall survival [26]. However, two additional studies revealed that HSP60 expression was connected to a considerably improved prognosis [29, 30]. Our results showed low HSPD1 expression was associated with unfavorable prognosis in all OC and serous OC. In addition, a normal human ovarian epithelial cell line (IOSE-80) had significant higher expression of mitochondrial HSP60 as compared to OC cell lines (SKOV3, OVCAR3, ES2), whereas A2780 showed no significant difference. Cell lines with different sources and genetic background may exhibit different biological behaviors. Cell lines with relatively low expression of HSP60 (SKOV3 and OVCAR3) were therefore selected for the rest of the study. We found HSP60-silencing in OC cells led to promotion of cell proliferation and migration in vitro. Metabolite analysis reinforced that HSP60 could be a factor contributing to tumor suppression in OC cells. These findings were inconsistent with the previous studies reporting inhibitory effects on cell proliferation and migration of OC cells (A2780 or TOV-21G) with HSP60-silencing [27, 28]. HSP60 knockdown stimulates the adenine-AMPK pathway, which inhibits the growth of OC cells mediated by the mTOR pathway. Adenine precursor 5′-methylthioadenosine phosphorylase (MTAP), which catalyzes the conversion of MTA to adenine, is upregulated in HSP60-KD A2780 cells [27]. HSP60 knockdown may inhibit the migration and proliferation of TOV-21G through downregulating aldehyde dehydrogenase 2 (ALDH2) and Acyl-CoA Dehydrogenase Short Chain (ACADS), which are all involved in the fatty acid degradation pathway [28]. Our current results suggested OXSM as the potential target of HSP60. Meanwhile, protein levels of MTAP didn’t show a significant change with HSP60-silencing. Most of the differentially expressed proteins enriched in the fatty acid-related pathways in the previous study showed different trends of changes in our study. The discrepancies might be due to different cell lines with different baseline characteristics.

One previous study found that there is a link between morphology and the origin of the 33 unique OC cell lines. SKOV3 and OVCAR3 have an epithelial morphology and are derived from ascitic fluid rather than primary tumor. A2780 and TOV21G have a round morphology, while ES2 have a spindle morphology. A2780, TOV21G and ES2 originated from tumor tissue [53]. Another study investigated cell cycle parameters, 2D migration, and 3D invasion behavior. Cell line doubling times were as follows: A2780 14 h, TOV21G 18 h, SKOV3 36 h, OVCAR3 47 h. In the characterization of cell cycle behavior, the percentage of cells in G0/G1 phase in SKOV3 cells is higher than A2780 and TOV21G while the percentage in S phase is significantly lower than A2780 and TOV21G. Analysis of the migration behavior shows A2780 migrates slower than the other NS cell lines, behaving more similarly to the HGS panel and OVCAR3 migrates significantly faster than the other HGS cell lines. In the characterization of invasive behavior, TOV21G and SKOV3 have greater invasive potential into matrigel, meanwhile, only TOV21G and A2780 can invade into the collagen matrix while SKOV3 can’t [54]. The cell morphology, origin and cell behavior are all related to specific gene profiles, which can explain the pro- and anti-oncogenic roles of signaling molecules and the cell type-specific effects of gene regulation.

In follow-up experiments, combined analysis of OXSM protein expression with or without MG132 treatment demonstrated that HSP60 could sustain the stability of OXSM. Overexpression of OXSM could reverse sh-HSPD1-mediated stimulation of proliferation. OXSM is one of the mitochondrial enzymes responsible for fatty acid synthesis in the mitochondria (mtFAS). MtFAS currently has one known product: lipoic acid. This important cofactor is required for the catalytic activity of a number of mitochondrial enzymes such as pyruvate dehydrogenase and a-ketoglutarate dehydrogenase [37]. To date there has been limited research on OXSM, which has shown that OXSM is associated with a poor prognosis in oral squamous cell patients and promotes the cell cycle [55] and inhibits apoptosis of glioma cells [56]. However, in our study, Kaplan-Meier survival analysis showed that low OXSM expression was associated with unfavorable prognosis in OC patients. OXSM downregulation markedly increased cell proliferation and migration. These results indicated that OXSM played a role in inhibiting ovarian cancer. However, the role of OXSM in ovarian cancer needs further exploration. Consistent with the potential tumor suppressive effect of OXSM in OC, LA exhibits a growth inhibitory effect and induces apoptosis in ovarian cell lines [57, 58]. LA exhibits a cytotoxic effect and is capable of suppressing growth of various cancer cell lines [59–62]. These effects have also been shown in vivo. LA has been used in syngenic mouse models with melanoma and bladder cancer cells [63] and mice inoculated with SkBr3 human breast cancer cells [64], which strongly delayed tumor growth and prolonged survival. LA’s growth-inhibitory effect in the ovarian cancer cells is linked to the lengthening of the half-life of the cyclin-dependent kinase inhibitor, p27kip1 [57] and downregulation of two anti-apoptotic proteins, Mcl-1 and Bcl-xL protein and a strong induction of the BH3-only protein Bim [58]. In order to investigate whether HSP60 regulated growth via LA, ovarian cancer stable cell lines were treated with LA. LA could reverse sh-HSPD1-mediated induction of proliferation. The results suggested that HSP60 inhibited the proliferation of OC cells via the LA synthesis pathway mediated by OXSM. However, further study is required to elucidate the specific role of HSP60 in different cellular contexts fully, as well as the correlation between HSP60 and OC with different histopathological phenotypes and pathological grades. In vivo studies are also necessary to verify the results of the present study.

## Conclusions

In summary, the decreased expression of HSP60 promoted proliferation and migration in SKOV3 and OVCAR3 cells. Suppression of HSP60 resulted in the downregulation of a series of mitochondrial proteins including OXSM, which was involved in lipid acid synthesis and metabolism. Additionally, we demonstrated that HSP60 interacted with OXSM and maintained its stability. These findings offer a more adequate theoretical foundation for comprehending the molecular pathology of ovarian cancer and provide the fundamental information needed to consider HSP60 as a potential diagnostic and therapeutic target for the disease.

## Electronic supplementary material

Below is the link to the electronic supplementary material.

**Supplementary Material 1**

**Supplementary Material 2**

**Supplementary Material 3**

**Supplementary Material 4**

**Supplementary Material 5**

**Supplementary Material 6**

## Data Availability

The data used and/or analysed during the current study are available from the corresponding author on reasonable request.
