# Correction to: Knockdown of heat shock protein family D member 1 (HSPD1) promotes proliferation and migration of ovarian cancer cells via disrupting the stability of mitochondrial 3-oxoacyl-ACP synthase (OXSM)

**DOI:** 10.1186/s13048-023-01184-4

**Published:** 2023-05-11

**Authors:** Yaoyun Duan, Juan Yu, Miaojuan Chen, Qinsheng Lu, Fen Ning, Xiaowen Gan, Hanbo Liu, Yixin Ye, Shenjiao Lu, Gendie E. Lash

**Affiliations:** 1 Division of Uterine Vascular Biology, Guangdong Provincial Clinical Research Center for Child Health, Guangzhou Institute of Pediatrics, Guangzhou Women and Children’s Medical Center, Guangzhou Medical University, Guangzhou, 510623 China; 2 School of Medicine, South China University of Technology, Guangzhou, China

**Correction: J Ovarian Res 16:81 (2023)**

10.1186/s13048-023-01156-8

Following publication of the original article [1], the authors identified an error on author Yixin Ye’s affiliation which is affiliation 1 instead of affiliation 2.

The original article has been corrected.
