# Supplementary material for: Knockdown of heat shock protein family D member 1 (HSPD1) promotes proliferation and migration of ovarian cancer cells via disrupting the stability of mitochondrial 3-oxoacyl-ACP synthase (OXSM)
Source: J Ovarian Res. 2023 Apr 22;16:81. doi: 10.1186/s13048-023-01156-8 (PMC10122320; doi:10.1186/s13048-023-01156-8)
Supplement: Supplementary file 1 — Supplementary Material 1 [file 13048_2023_1156_MOESM1_ESM.pdf]

ES-2 cells STR report

Method: An appropriate amount of ES-2 cells (Cell number XB0422-0225,1×10^6) were used Chelex100 to extract DNA, 20 STR loci and gender identification loci were amplified by 21 CELLID System, PCR product detection was performed by ABI3130x1 genetic analyzer, detection results were analyzed by GeneMapper IDX software (Applied Biosystems), and compared with ATCC, DSMZ, JCRB, Cellosaurus and other databases.

Experimental result:

1. The results of negative and positive control were correct.
2. The genotyping results of STR locus of ES-2 cell line is shown in the following table.

Conclusion:

1. The genomic DNA of ES-2 cell line is clear and the result of genotyping is good.
2. The results of STR typing showed that no cross contamination of human cell was found in the cell line of ES-2 cell line.
3. The DNA typing of the cell line was 100.00% matched with the cell type in the cell bank, and the cell line name was ES-2.

网站：www.procell.com.cn
电话：400-999-2100；027-87287608
邮箱：sales@procell.com.cn
地址：湖北省武汉市高新大道858号生物医药产业园三期C4栋

Appendix I: The genotyping results of STR locus of ES-2 cell line

STR Loci	Sample：XB0422-0225	Database：ES-2
Amelogenin	X,X	X
CSF1PO	10,15	10,15
D2S1338	17,23	17,23
D3S1358	15,18	15,18
D5S818	11,13	11,13
D7S820	11,11	11
D8S1179	14,14	14
D13S317	11,11	11
D16S539	11,13	11,13
D18S51	13,15	13,15
D19S433	15,15.2	15,15.2
D21S11	32.2,33.2	32.2,33.2
FGA	21,21	21
PentaD	8,13	8,13
PentaE	13,16	13,16
TH01	9.3,9.3	9.3
TPOX	8,12	8,12
vWA	16,17	16,17
D6S1043	11,12	
D12S391	20,21	
D2S411	11,11	
The Cellosaurus database has a matching rate of 100.00%，The number of matched bits is 17 （https://web.expasy.org/cellosaurus-str-search/）		

Note:

1. According to the cell STR identification standard established by the International Cell Line Authentication Committee (ICLAC), when the matching degree of cell lines is ≥80%, they are considered to be correlated, that is, derived from common ancestral cells; The matching degree is between 55% and 80%, and the correlation needs to be further verified. Less than 55% indicates no correlation between the two.
2. The effective peak of the map was the real PCR band; Small peaks and nonspecific bands were ignored in the calculation.

网站：www.procell.com.cn
电话：400-999-2100；027-87287608
邮箱：sales@procell.com.cn
地址：湖北省武汉市高新大道858号生物医药产业园三期C4栋

Appendix II: The genotyping results of STR locus of ES-2 cell line

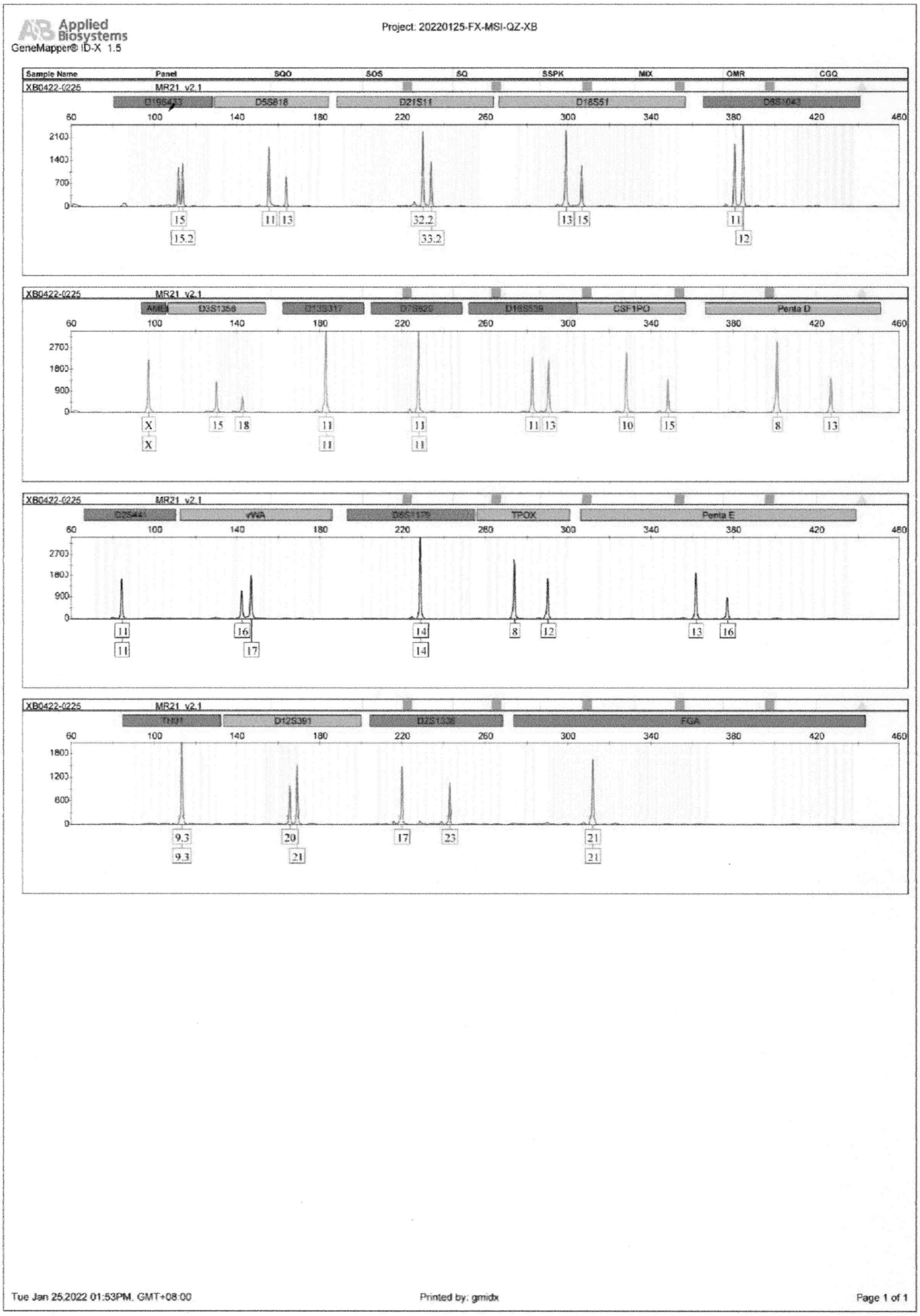

网站：www.procell.com.cn
电话：400-999-2100；027-87287608
邮箱：sales@procell.com.cn
地址：湖北省武汉市高新大道858号生物医药产业园三期C4栋
