# Supplementary material for: Knockdown of heat shock protein family D member 1 (HSPD1) promotes proliferation and migration of ovarian cancer cells via disrupting the stability of mitochondrial 3-oxoacyl-ACP synthase (OXSM)
Source: J Ovarian Res. 2023 Apr 22;16:81. doi: 10.1186/s13048-023-01156-8 (PMC10122320; doi:10.1186/s13048-023-01156-8)
Supplement: Supplementary file 2 — Supplementary Material 2 [file 13048_2023_1156_MOESM2_ESM.pdf]

# *NIH:OVCAR-3[OVCAR3]cells STR report*

**Method:** An appropriate amount of NIH:OVCAR-3 [OVCAR3] cells (Cell number PC-H2022062407,1×10$^6$) were used Microread Genomic DNA Kit to extract DNA, 20 STR loci and gender identification loci were amplified by Microreader$^{TM}$21 ID System, PCR product detection was performed by GenReader 7010 genetic analyzer, detection results were analyzed by GeneMapper Software6(Applied Biosystems), and compared with ATCC, DSMZ, JCRB, ExPASy and other databases.

**Experimental result:**

1. The results of negative and positive control were correct.
2. The genotyping results of STR locus of NIH:OVCAR-3 [OVCAR3] cell line is shown in the following table.

**Conclusion:**

1. The genomic DNA of NIH:OVCAR-3 [OVCAR3] cell line is clear and the result of genotyping is good.
2. The results of STR typing showed that no cross contamination of human cell was found in the cell line of NIH:OVCAR-3 [OVCAR3] cell line.
3. The DNA typing of the cell line was 96.55% matched with the cell type in the cell bank, and the cell line name was NIH:OVCAR-3 [OVCAR3].

**Appendix I: The genotyping results of STR locus of NIH:OVCAR-3 [OVACAR3]cell line.**

| **STR Loci** | **Sample：PC-H2022062407** | **Database：NIH:OVCAR-3 [OVCAR3]** |
|---|---|---|
| Amelogenin | X | X |
| CSF1PO | 11,12 | 11,12 |
| D2S1338 | 17,21 | 17,21 |
| D3S1358 | 17,18 | 17,18 |
| D5S818 | 11,12 | 11,12 |
| D7S820 | 10 | 10 |
| D8S1179 | 10,15 | 10,15 |
| D13S317 | 12 | 12 |
| D16S539 | 12 | 12 |
| D18S51 | 13 | 13 |
| D19S433 | 14,16.2 | 16.2 |
| D21S11 | 29,31.2 | 29,31.2 |
| FGA | 21 | 21 |
| PentaD | 12,13 | 12,13 |
| PentaE | 7,13 | 7,13 |
| TH01 | 9 | 9 |
| TPOX | 8 | 8 |
| vWA | 17 | 17 |
| D6S1043 | 10,11 | |
| D12S391 | 19,22 | 22 |
| D2S411 | 11,14 | 11,14 |
| The ExPASy database has a matching rate of 96.55%，The number of matched bits is 19 （https://web.expasy.org/cellosaurus-str-search/） | | |

**Note:**

1. According to the cell STR identification standard established by the International Cell Line Authentication Committee (ICLAC), when the matching degree of cell lines is ≥80%, they are considered to be correlated, that is, derived from common ancestral cells; The matching degree is between 55% and 80%, and the correlation needs to be further verified. Less than 55% indicates no correlation between the two.
2. The effective peak of the map was the real PCR band; Small peaks and nonspecific bands were ignored in the calculation.

网站：www.procell.com.cn
电话：400-999-2100；027-87287608
邮箱：sales@procell.com.cn
地址：湖北省武汉市高新大道858号生物医药产业园三期C4栋

**Appendix II: The genotyping results of STR locus of NIH:OVCAR-3 [OVCAR3] (Cell number PC-H2022062407) cell line.**

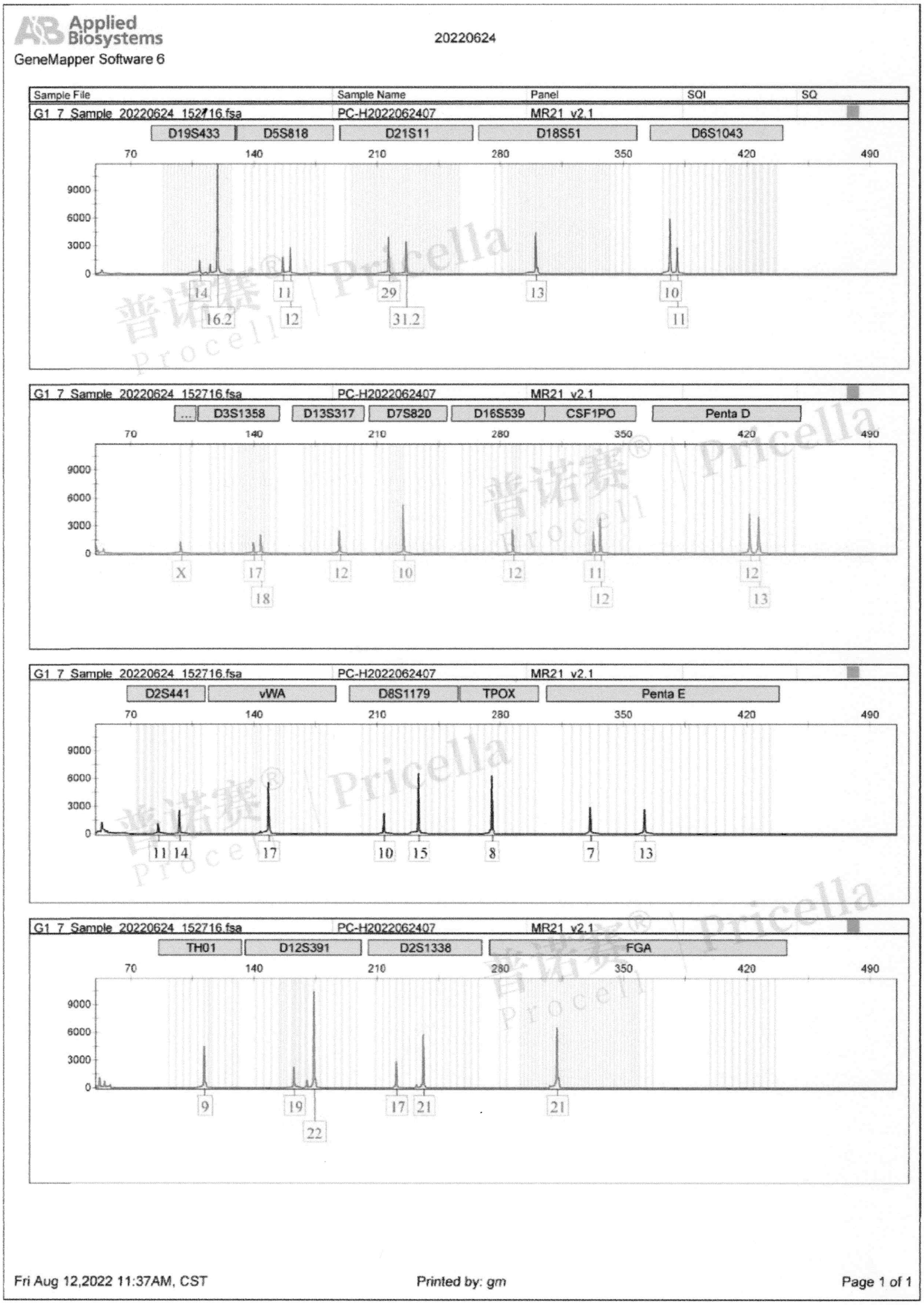


网站：www.procell.com.cn
电话：400-999-2100；027-87287608
邮箱：sales@procell.com.cn
地址：湖北省武汉市高新大道858号生物医药产业园三期C4栋
