# Supplementary material for: Knockdown of heat shock protein family D member 1 (HSPD1) promotes proliferation and migration of ovarian cancer cells via disrupting the stability of mitochondrial 3-oxoacyl-ACP synthase (OXSM)
Source: J Ovarian Res. 2023 Apr 22;16:81. doi: 10.1186/s13048-023-01156-8 (PMC10122320; doi:10.1186/s13048-023-01156-8)
Supplement: Supplementary file 3 — Supplementary Material 3 [file 13048_2023_1156_MOESM3_ESM.pdf]

Guangzhou jenniobio Biotechnoligy Co.,Ltd

Cell STR Certification Report

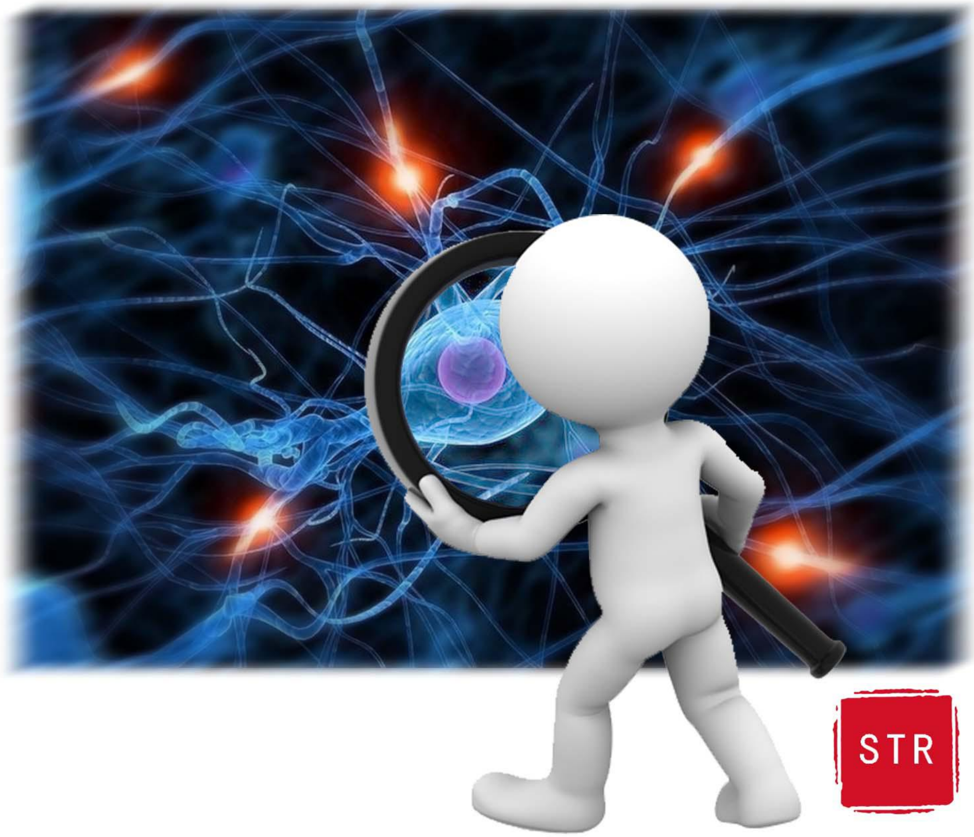

JENNIO Biological Technology

TEL: +86-020-29185636; +86-020-22154480

Address: 31 KeFeng Road, Luogang District, 510663, Guangzhou,China

Web: http://jennio-bio.com/

1、 Sample ID:A2780

2、 Original Material:Cell pellets

3、 Check time:2020-5-20

4、 Methods:

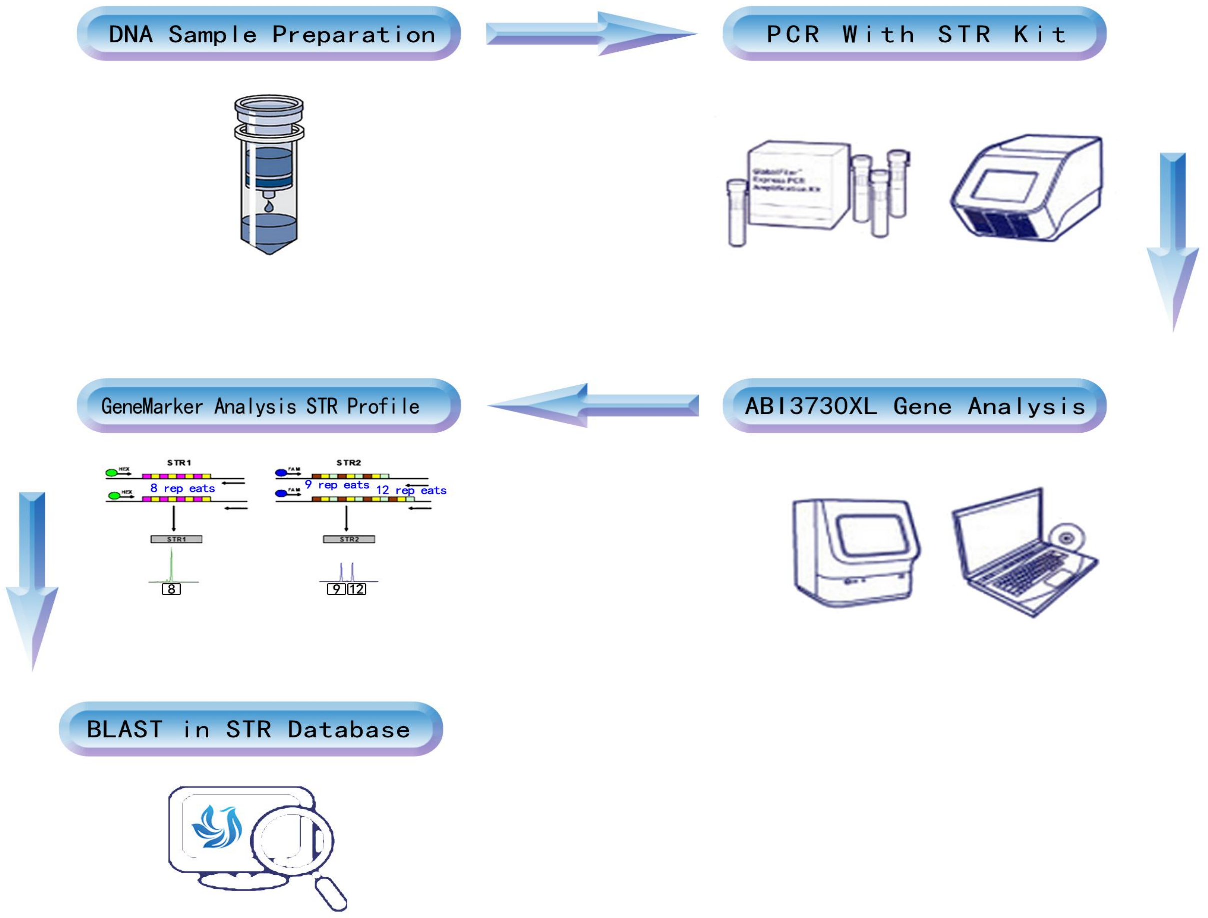

5、 Results：

Negative and positive test results are correct.

Amplification map of Genomic DNA clear， Genotyping results well.

STR Profile :

Genetic Site	Customer sample		ECACC	
	A2780		A2780	
Amelogenin	X	X	X	X
CSF1PO	10	11	10	11
D13S317	12	13	12	13
D16S539	11	13	11	13
D5S818	11	12	10	12
D7S820	10	10	10	10
THO1	6	6	6	6
TPOX	8	10	8	10
vWA	15	16	15	16

Percent match between the sample and the database profile：94.4

6、 Summary：

The result of STR profile showed no more than 2 distinct alleles were found ,the sample derived from a common ancestry(Figure 1); which matched 94.4**%** the reference cell line in the ECACC STR database, named A2780.

Notes：

- P=100% x (2xM)/N; M: number of the matching peaks; N: number of all peaks
  For example:M=17, N=36,P=100% x (2x17)/36=94.4%
- Based on ASN-0002-2011 Standard， cell lines with ⩾80% match are considered to be related ; i.e.,derived from a common ancestry. Cell lines with between a 55% to 80% match require futher profiling for authentication of relatedness.
- This data and analysis are for research use only.

Operator: Yuqiu Qin

Auditor: Jigang Tu

GUANGZHOU JENNIO BIOTECH CO.,LTD
22.May.2020
CELL STR PROFILING
PASS

Report time: 2020-5-22

Figure：

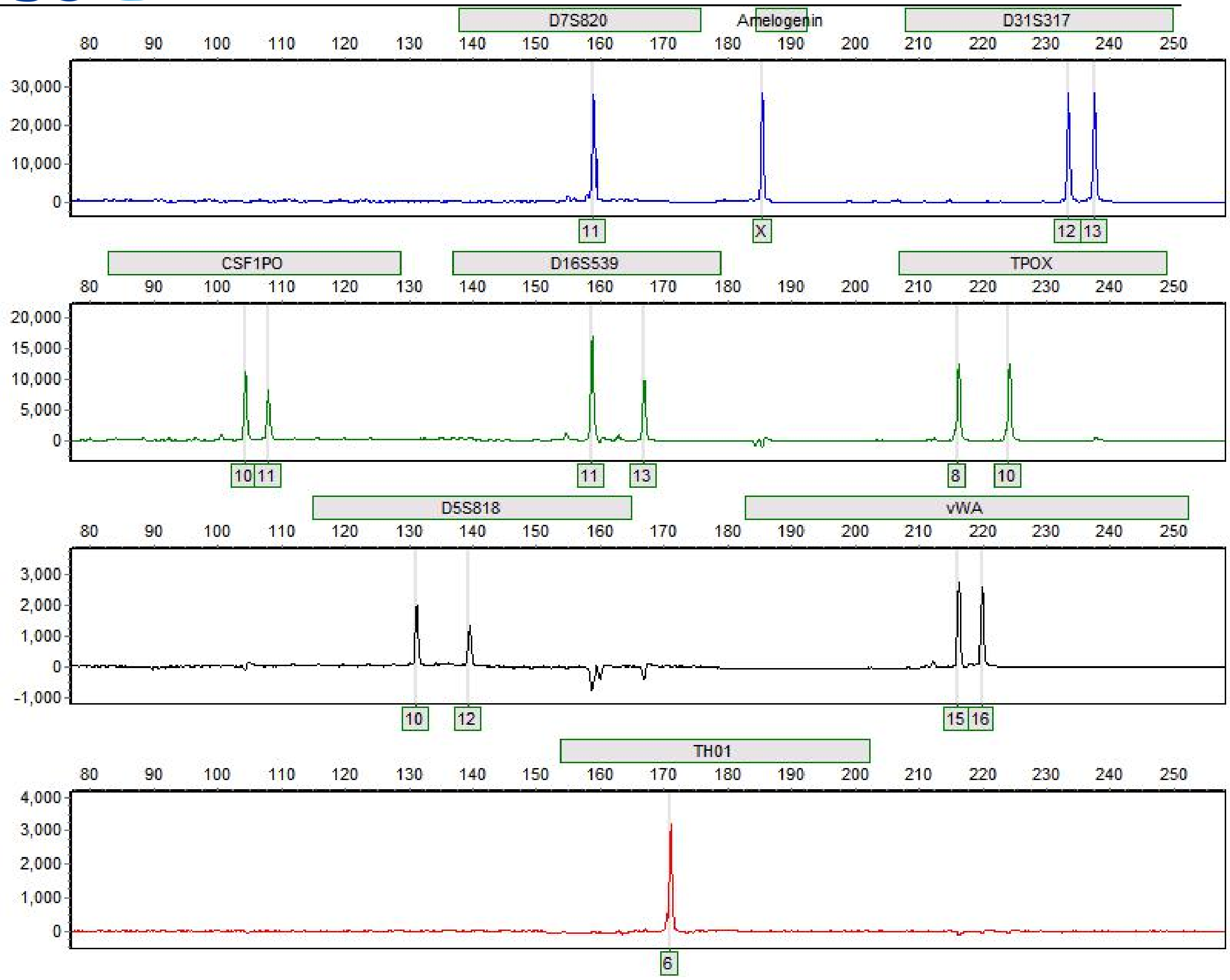

Figure 1.STR profiles of A2780 cell line
