# Supplementary material for: Knockdown of heat shock protein family D member 1 (HSPD1) promotes proliferation and migration of ovarian cancer cells via disrupting the stability of mitochondrial 3-oxoacyl-ACP synthase (OXSM)
Source: J Ovarian Res. 2023 Apr 22;16:81. doi: 10.1186/s13048-023-01156-8 (PMC10122320; doi:10.1186/s13048-023-01156-8)
Supplement: Supplementary file 4 — Supplementary Material 4 [file 13048_2023_1156_MOESM4_ESM.pdf]

Guangzhou jenniobio Biotechnoligy Co.,Ltd

Cell STR Certification Report

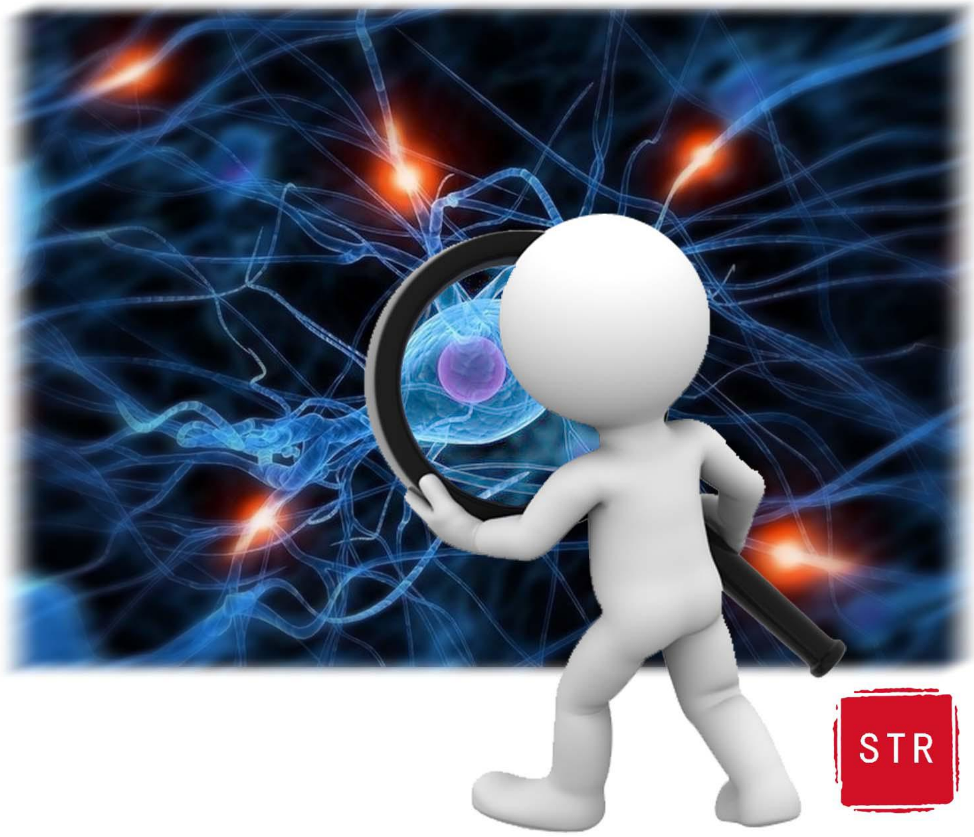

JENNIO Biological Technology
TEL: +86-020-29185636; +86-020-22154480
Address: 31 KeFeng Road, Luogang District, 510663, Guangzhou,China
Web: http://jennio-bio.com/

1、 Sample ID：SK-OV-3

2、 Original Material：Cell pellets

3、 Check time:2021-7-13

4、 Methods：

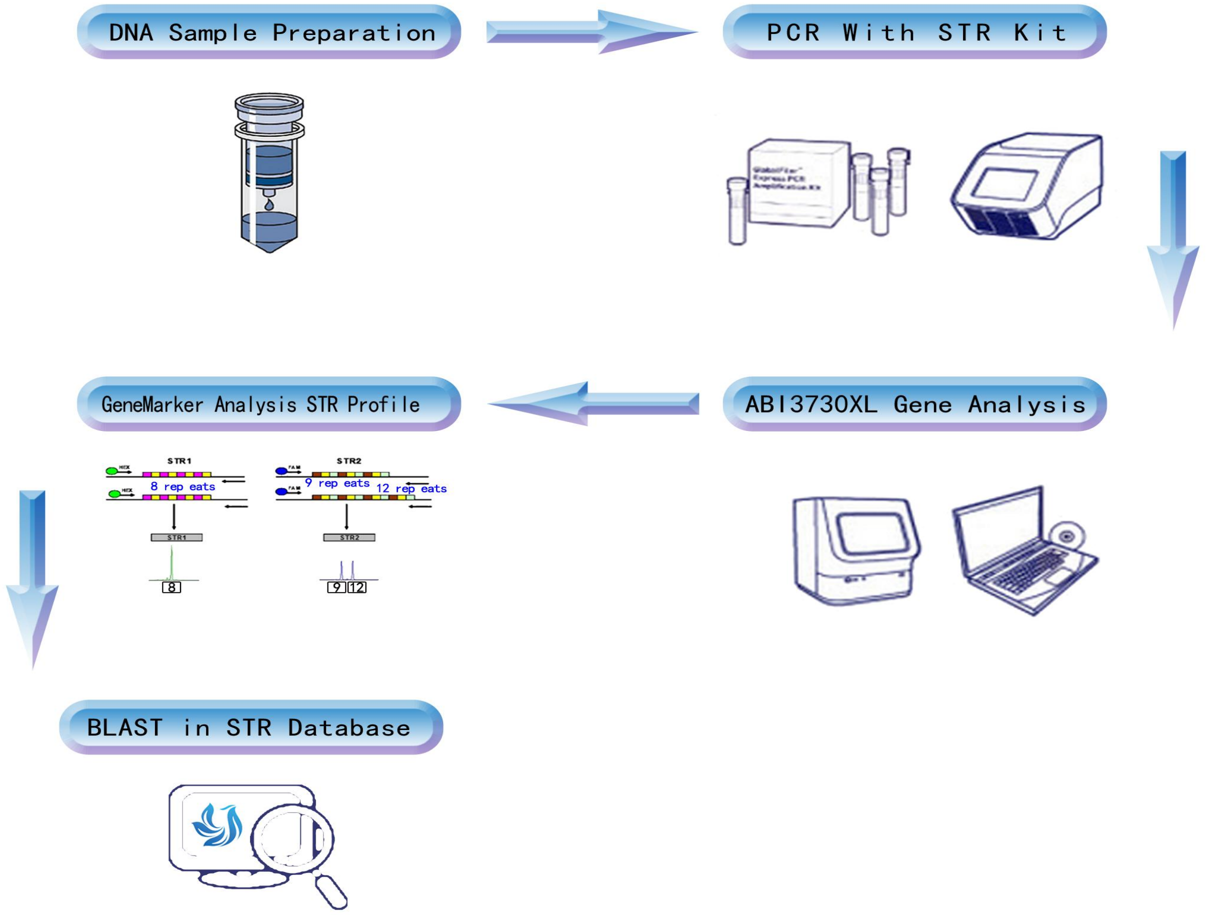

5、 Results：

Negative and positive test results are correct.

Amplification map of Genomic DNA clear，Genotyping results well.

STR Profile :

Genetic Site	Customer sample		**ATCC**	
	SK-OV-3		SK-OV-3	
Amelogenin	X	X	X	X
CSF1PO	11	11	11	11
D13S317	8	11	8	11
D16S539	12	12	12	12
D5S818	11	11	11	11
D7S820	13	14	13	14
THO1	9	9.3	9	9.3
TPOX	8	11	8	11
vWA	17	18	17	18

Percent match between the sample and the database profile: **100%**

6、 Summary:

The result of STR profile showed no more than 2 distinct alleles were found ,the sample derived from a common ancestry(Figure 1); which matched **100%** the reference cell line in the ATCC STR database, named SK-OV-3 .

Notes:

- P=100% x (2xM)/N; M=18, N=36,P=100% x (2x18)/36=100%
  M: number of the matching peaks; N: number of all peaks
- Based on ASN-0002-2011 Standard, cell lines with ⩾80% match are considered to be related; i.e.,derived from a common ancestry. Cell lines with between a 55% to 80% match require futher profiling for authentication of relatedness.
- This data and analysis are for research use only.

Operator: Yuqiu Qin

GUANGZHOU JENNIO BIOTECH CO.,LTD
15 Jul. 2021
CELL STR PROFILING
PASS

Auditor: Jigang Tu

Report time: 2021-7-15

Figure:

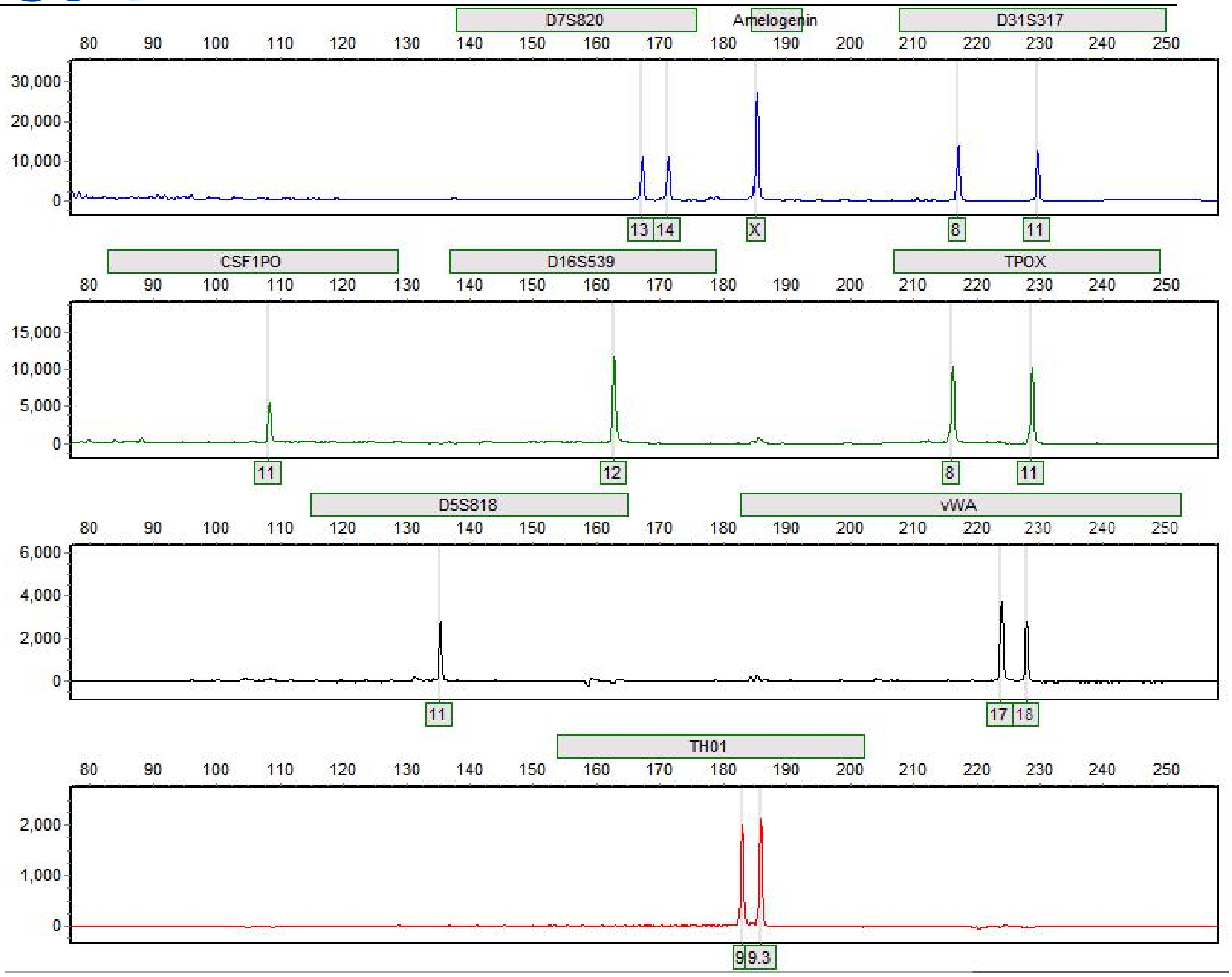

Figure 1.STR profiles of SK-OV-3 cell line
