# Supplementary material for: Knockdown of heat shock protein family D member 1 (HSPD1) promotes proliferation and migration of ovarian cancer cells via disrupting the stability of mitochondrial 3-oxoacyl-ACP synthase (OXSM)
Source: J Ovarian Res. 2023 Apr 22;16:81. doi: 10.1186/s13048-023-01156-8 (PMC10122320; doi:10.1186/s13048-023-01156-8)
Supplement: Supplementary file 5 — Supplementary Material 5 [file 13048_2023_1156_MOESM5_ESM.doc]

**Supplementary Figure 1**

**
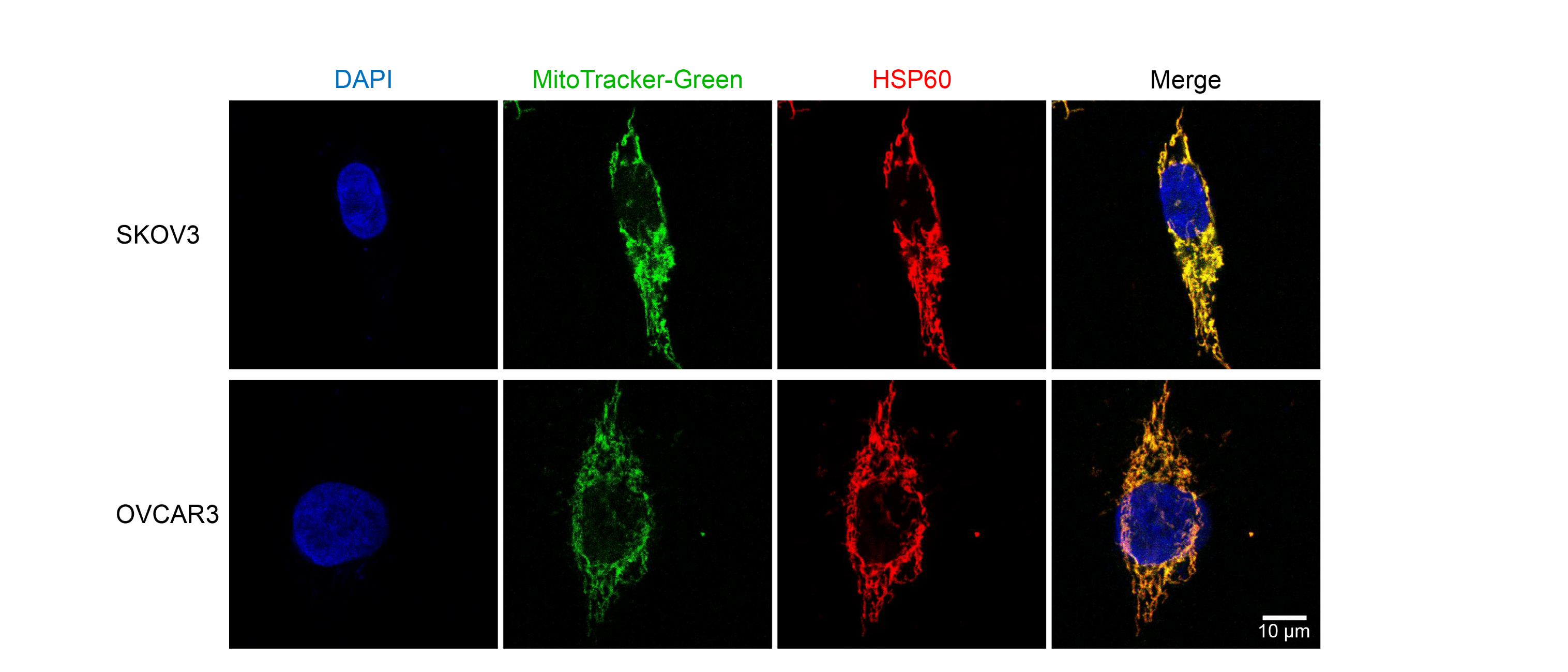
**

**Supplementary Fig. 1 Immunofluorescence images depicting the mitochondrial localization of HSP60 in SKOV3 and OVCAR3 cells.** HSP60 appeared red and MitoTracker appeared green. The colocalization of HSP60 and MitoTracker indicated the mitochondrial localization. Scale bar = 10 μm.

**Supplementary Figure 2**


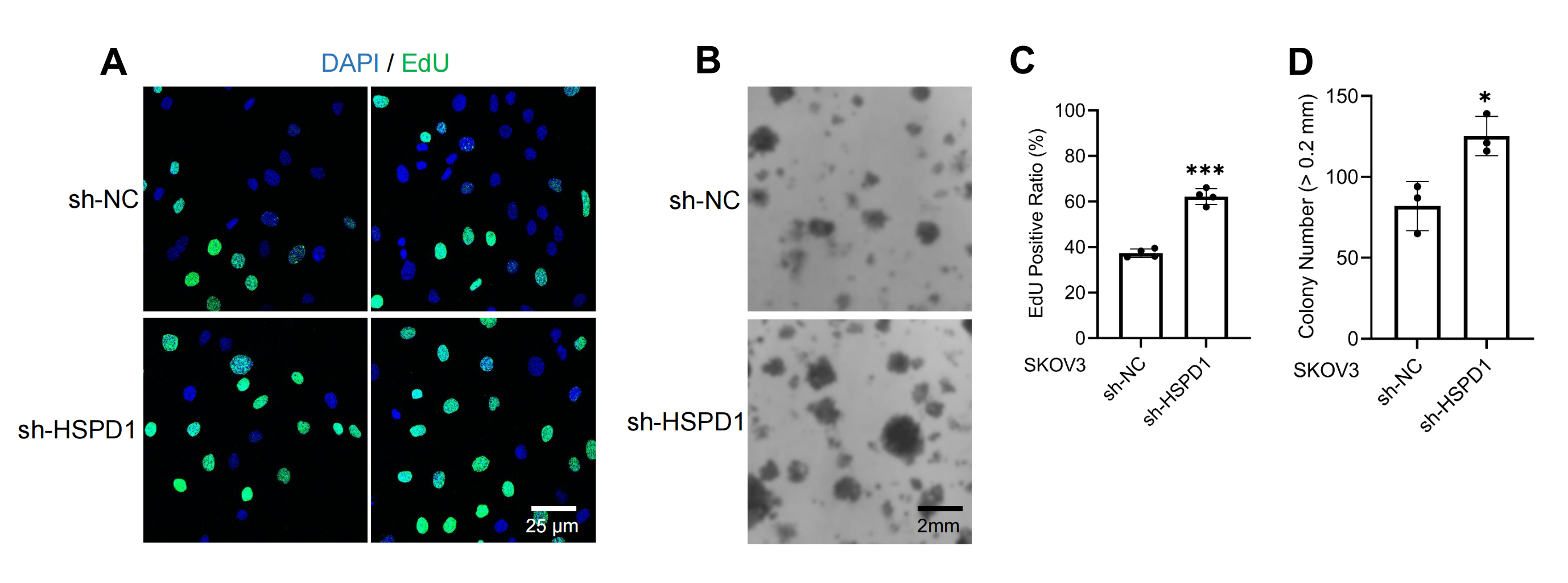


**Supplementary Fig. 2 Promoted tumor cell proliferation in HSPD1 knockdown SKOV3 cells. A** The EdU incorporation assay was performed in SKOV3 cells. Scale bar = 25 μm. **B** Soft agar colony formation assay of SKOV3 cells. Scale bar = 2mm. **C** EdU positive ratio was calculated as the ratio of positive cells to total cells. n = 3. **D** Colony number was counted in the soft agar assay. n = 3. *P<0.05, ***P<0.001.

**Supplementary Figure 3**

**
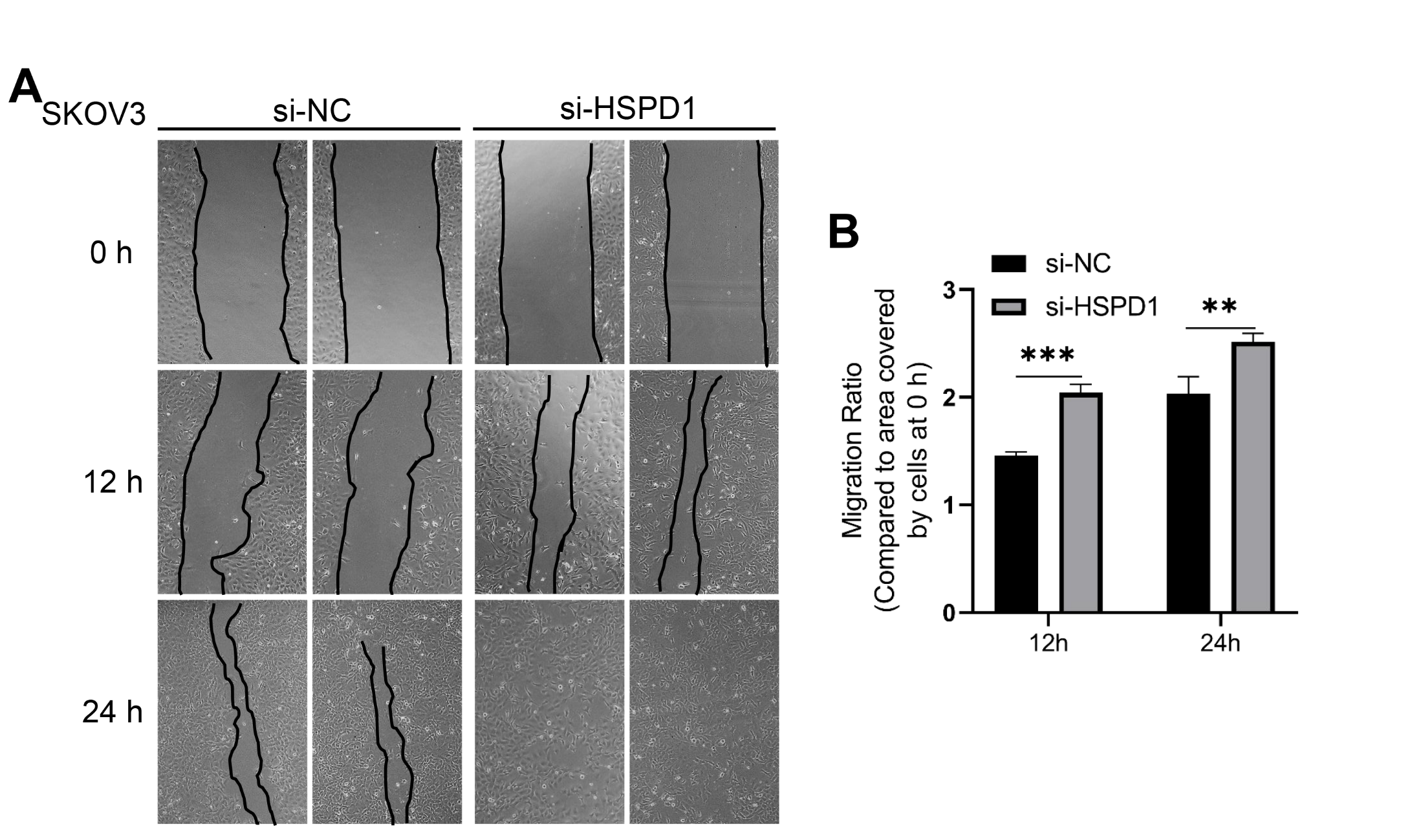
**

**Supplementary Fig. 3 Knockdown of HSPD1 promoted ovarian cancer cell migration in vitro. A** Cell migration was measured by the wound healing assay in SKOV3 cells transfected with si-HSPD1 and si-NC. **B** Quantification analysis of the influence of HSP60 on cell migration with the wound healing assay. n = 4, ***P*<0.01, ****P*<0.001.

**Supplementary Table. 1** The list of detected proteins through proteomic analysis.
